# Noncoding RNAs: an emerging modulator of drug resistance in pancreatic cancer

**DOI:** 10.3389/fcell.2023.1226639

**Published:** 2023-07-25

**Authors:** Ling Wei, Jujie Sun, Xingwu Wang, Yizhou Huang, Linying Huang, Linyu Han, Yanxiu Zheng, Yuan Xu, Nasha Zhang, Ming Yang

**Affiliations:** ^1^ Shandong Provincial Key Laboratory of Radiation Oncology, Cancer Research Center, Shandong Cancer Hospital and Institute, Shandong First Medical University and Shandong Academy of Medical Sciences, Jinan, Shandong, China; ^2^ Department of Pathology, Shandong Cancer Hospital and Institute, Shandong First Medical University and Shandong Academy of Medical Sciences, Jinan, Shandong, China; ^3^ Department of Radiation Oncology, Shandong Cancer Hospital and Institute, Shandong First Medical University and Shandong Academy of Medical Sciences, Jinan, Shandong, China; ^4^ Jiangsu Key Lab of Cancer Biomarkers, Prevention and Treatment, Collaborative Innovation Center for Cancer Personalized Medicine, Nanjing Medical University, Nanjing, Jiangsu, China

**Keywords:** pancreatic cancer, drug resistance, microRNA, long non-coding RNA, circular RNA

## Abstract

Pancreatic cancer is the eighth leading cause of cancer-related deaths worldwide. Chemotherapy including gemcitabine, 5-fluorouracil, adriamycin and cisplatin, immunotherapy with immune checkpoint inhibitors and targeted therapy have been demonstrated to significantly improve prognosis of pancreatic cancer patients with advanced diseases. However, most patients developed drug resistance to these therapeutic agents, which leading to shortened patient survival. The detailed molecular mechanisms contributing to pancreatic cancer drug resistance remain largely unclear. The growing evidences have shown that noncoding RNAs (ncRNAs), including microRNAs (miRNAs), long noncoding RNAs (lncRNAs) and circular RNAs (circRNAs), are involved in pancreatic cancer pathogenesis and development of drug resistance. In the present review, we systematically summarized the new insight on of various miRNAs, lncRNAs and circRNAs on drug resistance of pancreatic cancer. These results demonstrated that targeting the tumor-specific ncRNA may provide novel options for pancreatic cancer treatments.

## Introduction

Pancreatic cancer (PC), a highly lethal human malignancy, ranks the fourth leading cause of cancer-related death in the United States and the eighth worldwide. PC patients had only 5% overall survival rate at 5-years after diagnosis (Von Hoff et al., 2009). A total of 458,918PC cases were diagnosed all around the world and 432,242 deaths were reported in 2018 (Bray et al., 2018). Pancreatic ductal adenocarcinoma (PDAC) is the most common pathological type, accounting for 85%–90% of all PC patients. Due to lack of the effectively early diagnosis methods, the vast majority of PC patients are diagnosed at advanced disease stages and tumors are unresectable. Systematical therapy with chemotherapy, immunotherapy and targeted therapy drugs with or without radiation therapy is only viable alternative for advanced PC patients. Currently, chemotherapeutic agents applied in PC treatments include gemcitabine (GEM), 5-fluorouracil (5-FU), adriamycin (ADM), cisplatin (DDP), oxaliplatin, nab-paclitaxel, irinotecan and capecitabine (Springfeld et al., 2019). Targeted therapy drugs for PC include small molecule tyrosine kinase inhibitor (TKI) erlotinib targeting epidermal growth factor receptor (EGFR), oral mammalian target of rapamycin (mTOR) inhibitor everolimus and small molecule multi-target receptor TKI sunitinib which targets RTK. GEM combined with erlotinib is commonly used to treat locally advanced PC or metastatic PC (Irigoyen et al., 2017; Halfdanarson et al., 2019). Everolimus and sunitinib were applied in PC patients with progressive neuroendocrine tumors that cannot be resected or have metastatic spread (Angelousi et al., 2017; Nunez et al., 2019). In the aspect of immunotherapy, there are several monoclonal antibodies of immune checkpoint inhibitors (ICIs), such as ICIs of cytotoxic T lymphocyte antigen-4 (CTLA-4) (ipilimumab and trimetamumab), as well as ICIs of programmed cell death protein 1(PD-1) (pidilizumab, nivolumab and pembrolizumab) (Weiss et al., 2018; Kamath et al., 2020). Unfortunately, intrinsic drug resistance or acquired drug resistance severely limits the applications of these therapeutic agents. The deregulations of cell cycle control, apoptosis, DNA damage repair, autophagy, epithelial-mesenchymal transition (EMT), ABC transporters and cancer stem cells (CSCs), have been reported to be associated with drug resistance in PC (Modi et al., 2016; Kuwada et al., 2018; Liu Q G et al., 2018; Ma T et al., 2018; Knudsen et al., 2019; Qian et al., 2019; Yang Z et al., 2020). Up to now, the molecular mechanisms for development of drug resistance in PC still remain largely unclear.

Noncoding RNAs (ncRNAs) transcripts, a series of RNA molecules lacking of proteins coding potentials, constitute about 70% of human genome and modulate most signaling pathways, physiological and pathological processes (Jadeja et al., 2020; Song et al., 2020; Su et al., 2020; Wang et al., 2020; Wei et al., 2020). Multiple types of ncRNAs have been identified, such as microRNAs (miRNAs), long ncRNAs (lncRNAs), circular RNAs (circRNAs), small nucleolar RNAs (snoRNAs) and PIWI-interacting RNAs (piRNAs) (Huang et al., 2020; Pammer et al., 2020; Qin et al., 2020; Xu W et al., 2020; Zhang Y et al., 2020; Zhao et al., 2020). A number of miRNAs, lncRNAs and circRNAs have been shown to be related to various cell behaviors, such as cell growth, apoptosis, cell cycle progression, EMT and autophagy (Luo et al., 2023; Xu, 2023). It has been demonstrated that many dysregulated ncRNAs in PC not only might serve as biomarkers of diagnosis and prognosis, but also contribute to resistance development to therapeutic agents and irradiation (Hao et al., 2019; Xu et al., 2019; Li et al., 2020b; Franses et al., 2020; Nguyen et al., 2020; Ye et al., 2020; Yu et al., 2020). Although a large number of ncRNAs have been found to be involved in PC chemoresistance, only a few ncRNAs were reported to confer resistance to targeted therapy in PC. miR-142-5p has been shown to modulate the expression of PD-L1 in PC cells and promote anti-tumor immunity (Jia et al., 2017), nevertheless, no studies have reported the impacts of ncRNAs on immunotherapy resistance of PC cells.

Considering the critical and complicated roles of ncRNAs in PC drug resistance development, herein, we systematically summarized the underlying molecular mechanisms how miRNAs, lncRNAs and circRNAs confer drug resistance in PC.

## miRNAs and drug resistance

miRNAs, a subclass of sncRNAs, negatively regulate target gene expression through binding to the 3′-untranslated region (3′-UTR) of the target mRNA and are involved in multiple cellular process, such as cell difference, proliferation, apoptosis, cell cycle progression, angiogenesis, EMT and CSC formation. Several dysregulated miRNAs, functioning as oncogenes or tumor suppressors, have been identified to be involved in resistance development to therapeutic agents in PC (Li et al., 2020a; An and Zheng, 2020; Guo et al., 2020; Jiang et al., 2020; Wu et al., 2020). Compared with naive cells, some abnormally expressed miRNAs have been reported in various drug-resistant PC cells (Dhayat et al., 2015; Shen et al., 2015; Tian et al., 2016). The involvement of miRNAs in PC resistance to GEM, 5-FU and other drugs are summarized below.

### MiRNAs and GEM resistance

GEM, a kind of cytosine nucleoside derivative, is currently the first-line standard chemotherapeutic agent to treat PC patients. However, only a few PC patients can maintain sensitivity to GEM chemotherapy and subsequently, inevitable drug resistance often leads to low response rate and poor treatment efficacy. A variety of miRNAs have been found to be associated with GEM resistance in PC. Multiple mechanisms, such as diminished apoptosis, increased DNA repair, disordered cell cycle, decreased intracellular drug accumulation due to increased expression of drug efflux transporters, EMT, as well as CSCs, play crucial roles in miRNAs-mediated development of GEM resistance in PC. Oncogenic and tumor suppressor miRNAs involved in GEM resistance are summarized in details (Table 1; Table 2).

**TABLE 1:** Oncogenic miRNAs modulating gemcitabine resistance in pancreatic cancer.

miRNAs	Expression^a^	Genes and pathways	References
miR-17-5p	upregulated	Bim	Yan et al. (2012)
miR-29a	upregulated	Dkk1/Kremen2/sFRP2/Wnt/β-catenin	Nagano et al. (2013)
miR-21	upregulated	PTEN/RECK	Park et al. (2009)
		PDCD4	Bhatti et al. (2011)
		Bcl-2	Dong et al. (2011)
		FasL	Wang et al. (2013)
		MMP-2/MMP-9/VEGF	Giovannetti et al. (2010)
		MMP-3/MMP-9/PDGF/CCL-7/PDCD4	Zhang L et al. (2018)
miR-365	upregulated	SHC1/BAX	Hamada et al. (2014)
		cytidine deaminase	Binenbaum et al. (2018)
miR-181b	upregulated	Bcl-2	Cai et al. (2013)
		NF-κB/CYLD	Takiuchi et al. (2013)
miR-181c	upregulated	Hippo signaling	Chen et al. (2015)
miR-155	upregulated	-	Mikamori et al. (2017)
		CAT, DCK	Patel et al. (2017)
miR-106b	upregulated	TP53INP1	Fang et al. (2019)
miR-210	upregulated	mTOR	Yang Q et al. (2020)
miR-301	upregulated	CDH1	Funamizu et al. (2019)
miR-301a-3p	upregulated	PTEN	Xia et al. (2017)
miR-301a	upregulated	TAp63	Luo et al. (2018)
miR-296-5p	upregulated	BOK/vimentin/N-cadherin	Okazaki et al. (2020)
miR-1246	upregulated	CCNG2/CSC	Hasegawa et al. (2014)
miR-125a	upregulated	A20	Yao et al. (2016)
miR-10a-5p	upregulated	TFAP2C	Xiong et al. (2018)
miR-342-3p	upregulated	KLF6	Ma et al. (2019)
miR-744	upregulated	-	Miyamae et al. (2015)
miR-135b	upregulated	BMAL1/YY1	Jiang et al. (2018)
miR-320c	upregulated	SMARCC1	Iwagami et al. (2013)
miR-93-5p	upregulated	PTEN/PI3K/Akt	Wu et al. (2021)
miR-331-3p	upregulated	Wnt/β-Catenin, ST7L	Zhan et al. (2020)
miR-3178	upregulated	RhoB/PI3K/Akt, ABC transporters	Gu et al. (2022)

^a^ miRNAs, upregulated in gemcitabine resistant pancreatic cancer cells. This table shows 23 miRNAs, whose expression levels and potential targets in gemcitabine resistance of pancreatic cancer.

**TABLE 2:** Tumor suppressive miRNAs modulating gemcitabine resistance in pancreatic cancer.

miRNAs	Expression^a^	Genes and pathways	References
miR-33a	downregulated	Pim-3/Akt/GSK-3β/β-catenin	Liang et al. (2015b)
		β-catenin	Liang et al. (2015a)
miR-497	downregulated	FGF2/FGFR1	Xu et al. (2014)
miR-210	downregulated	ABCC5	Amponsah et al. (2017)
miR-1285	downregulated	YAP1/EGFR/β-catenin	Huang et al. (2017)
miR-608	downregulated	RRM1/CDA	Rajabpour et al. (2017)
miR-506	downregulated	SPHK1/Akt/NF-κB	Li et al. (2016)
miR-30a-5p	downregulated	FOXD1/ERK	Zhou et al. (2019)
miR-146a-5p	downregulated	TRAF6/NF-kBp65/P-gp	Meng et al. (2020)
miR-34a	downregulated	Bcl-2/Notch1/Notch2	Ji et al. (2009)
miR-34	downregulated	Slug/PUMA	Zhang Q A et al. (2018)
miR-494	downregulated	c-Myc/SIRT1	Liu et al. (2015)
miR-373-3p	downregulated	CCND2	Hu et al. (2018)
miR-101	downregulated	DNA-dependent protein kinase catalytic subunit (DNA-PKcs)	Hu et al. (2017)
miR-101-3p	downregulated	RRM1	Fan et al. (2016)
miR-153	downregulated	Snail	Liu et al. (2017)
miR-374b-5p	downregulated	bcl-2	Sun et al. (2018)
miR-410-3p	downregulated	HMGB1	Xiong et al. (2017)
miR-29c	downregulated	USP22	Huang et al. (2018)
miR-127	downregulated	CCNE1, CDKN1A, CDKN1B, CCND1, CDK2	Panebianco et al. (2021)
miR-509-5p	downregulated	E-cadherin/ZO-1/ZEB1/Snail	Hiramoto et al. (2017)
miR-1243	downregulated	E-cadherin/ZO-1/ZEB1/Snail	Hiramoto et al. (2017)
miR-200c	downregulated	CSCs	Ma et al. (2015)
miR-200b	downregulated	ZEB1/ZEB2/CDH1	Funamizu et al. (2019)
miR-125a-3p	downregulated	Fyn	Liu B et al. (2018)
miR-3656	downregulated	RHOF	Yang R M et al. (2017)
miR-17-92cluster	downregulated	NODAL/ACTIVIN/TGF-β1/p21/p57/TBX3	Cioffi et al. (2015)
miR-205	downregulated	TUBB3/RRM1/ZEB1	Chaudhary et al. (2017)
miR-497	downregulated	NF-κB1	Yu Y et al. (2022)
miR-142-5p	downregulated	-	Ohuchida et al. (2011)
miR-145	downregulated	p70S6K1	Lin et al. (2016)
miR-429	downregulated	PDCD4	Yu et al. (2017)
miR-30a	downregulated	SNAI1/IRS1/ERK/AKT	Wang T et al. (2019)
miR-760	downregulated	MOV10/ITGB1	Yang et al. (2019)
let-7	downregulated	RRM2	Bhutia et al. (2013)
miR-211	downregulated	RRM2	Maftouh et al. (2014)
miR-20a-5p	downregulated	RRM2	Lu et al. (2019)
miR-7	downregulated	PARP1/NF-κB	Ye et al. (2021)
miR-136-5p	downregulated	ZNF32	Xu Y et al. (2020)
miR-3662	downregulated	HIF-1ɑ, glycolysis	Liu S L et al. (2021)

^a^ miRNAs, downregulated in gemcitabine resistant pancreatic cancer cells. This table shows 39 miRNAs, whose expression levels and potential targets in gemcitabine resistance of pancreatic cancer.

### Oncogenic miRNAs and GEM resistance

Several miRNAs, such as miR-17-5p, miR-21, miR-29a, miR-365, miR-155 and miR-181, have been found to confer GEM resistance through promoting proliferation and reducing apoptosis of PC cells. For example, silencing of oncogenic miR-17-5p, which targeting *Bim*, could potentiate GEM sensitivities, activate caspase-3 and promote apoptosis in human PANC-1 and BxPC3 PC cell lines (Yan et al., 2012). Similarly, inhibition of miR-29a could increase apoptotic cells, upregulate S phase fraction, and reverse GEM resistance via elevating expression of *Dikkopf-1* (*Dkk1*), *Kremen2*, *secreted frizzled related protein 2* (*sFRP2*) and activating the Wnt/β-catenin signaling in human MIAPaCa-2 and PSN-1 PC cells (Nagano et al., 2013). miR-21, which has been found to be upregulated in several cancers, could confer GEM resistance in PC by affecting expression of multiple target genes, including *phosphatase and tensin homologue deleted on chromosome ten* (*PTEN*)/*RECK*, *programmed cell death 4* (*PDCD4*), *Bcl-2*, *FasL*, *matrix metalloproteinase-2* (*MMP-2*), *MMP-9* and *VEGF* (Park et al., 2009; Giovannetti et al., 2010; Bhatti et al., 2011; Dong et al., 2011; Wang et al., 2013). Interestingly, patients with GEM-resistance PDAC demonstrated highly activated cancer-associated fibroblasts (CAFs) and elevated miR-21 expression. Overexpression of miR-21 in CAFs could significantly promote GEM resistance in PDAC. On the contrary, silencing of miR-21 in CAFs diminished GEM resistance. Meanwhile, CAFs with high miR-21 level also displayed increased expression of *platelet-derived growth factor* (*PDGF*), *MMP-3*, *MMP-9* and *chemokine (C-C motif) ligand 7* (*CCL7*) (Zhang L et al., 2018). Through directly suppressing expression of the apoptosis-promoting protein *BAX* and adaptor protein *Src Homology 2 Domain Containing 1* (*SHC1*), miR-365 has been shown to potentiate the resistance against GEM in PC cells (Hamada et al., 2014). Moreover, overexpressed miR-365 in macrophage-derived exosomes (MDE) could also contribute to GEM resistance through increasing the triphospho-nucleotide pool and enzyme cytidine deaminase in PDAC cells (Binenbaum et al., 2018). In addition, oncogenic miR-181 has also been shown to promote GEM resistance of PC cells via modulating expression of *Bcl-2*, *nuclear factor kappa B (NF-κB)* and *cylindromatosis* (*CYLD*) as well as activation of the Hippo signaling (Cai et al., 2013; Takiuchi et al., 2013; Chen et al., 2015). Interestingly, accumulating data has shown that exosomes play a role in the development of chemoresistance. Mikamori et al. found that miR-155, an over-expressed miRNA in GEM-resistant PANC-1-GR PDAC cells, could potentiate exosome secretion and confer GEM resistance in PDAC via anti-apoptosis effects. On the contrary, blocking exosome delivery could alleviate miR-155 induced GEM resistance (Mikamori et al., 2017). In addition, through inhibiting the expression of deoxycytidine kinase (DCK), an important enzyme involved in GEM metabolism, miR-155 could promote exosome-mediated acquired resistance to GEM in PC cells (Patel et al., 2017). Recently, it has been found that exosomal miR-106b deriving from CAFs, could confer GEM resistance through targeting *TP53INP1* in PC (Fang et al., 2019). By contrast, exosomes deriving from pancreatic CSCs with GEM resistance could deliver miR-210 and transform GEM-sensitive cells to drug-resistance cells via activating the *mTOR* signaling pathway (Yang Q et al., 2020).

EMT and CSCs also play crucial roles in development of drug resistance. It has been shown that several miRNAs conferred GEM resistance of PC via modulating EMT and CSCs. miR-301, a highly expressed miRNA in GEM-resistant Capan-2 and PANC-1 cells, could trigger EMT and potentiate GEM resistance through inhibiting *cadherin 1* (*CDH1*) expression (Funamizu et al., 2019). miR-301a-3p has also been shown to confer GEM resistance via suppression of *PTEN* expression (Xia et al., 2017). Hypoxia, a prevalent phenomenon during tumorigenesis, has been found to promote resistance of chemotherapy and radiotherapy in PC. miR-301a, a hypoxia-sensitive miRNA, has been shown to be involved in hypoxia-induced GEM resistance via targeting P63 family member *TAp63* in PC (Luo et al., 2018). In addition, through suppressing a pro-apoptotic gene of *Bcl2-related ovarian killer* (*BOK*) and EMT marker *vimentin* and *N-cadherin*, exogenous expression of miR-296-5p has been found to weaken the apoptosis induced by GEM, indicating that targeting miR-296-5p may have therapeutic potential to overcome GEM resistance of PC patients (Okazaki et al., 2020). Ectopic expression of miR-1246 could promote CSC-like properties and GEM resistance via inhibiting expression of *CCNG2*. Moreover, high expression levels of miR-1246 in PC tissues predicted poor prognosis of patients (Hasegawa et al., 2014).

In addition, oncogenic miR-125a, miR-10a-5p and miR-342-3p have also been found to enhance GEM resistance of PC cells through targeting A20, transcription factor activating protein 2 gamma (TFAP2C) and Krüppel-like factor 6 (KLF6), respectively (Yao et al., 2016; Xiong et al., 2018; Ma et al., 2019). Plasma miR-744 might be a valuable marker to predict poor prognosis and GEM resistance through PC patients’ plasma miRNA profiling analyses (Miyamae et al., 2015). Interestingly, a novel miR-135b-BMAL1-YY1 signaling, which could promote tumorigenesis and GEM resistance in pancreas, has been identified (Jiang et al., 2018). In addition, miR-320c could confer GEM resistance via modulating expression of SMARCC1, a core subunit of chromatin remodeling complex of switch/sucrose nonfermentable (SWI/SNF) in PC cells (Iwagami et al., 2013). Also, miR-93-5p could promote GEM resistance in PC cells through silencing expression of its target gene *PTEN* and, thus activating the PI3K/Akt pathway. miR-331-3p could confer GEM resistance in PC cells through targeting ST7L and activating the Wnt/β-Catenin signaling (Zhan et al., 2020; Wu et al., 2021). miR-3178 has also been found to promote GEM resistance via activating the RhoB/PI3K/Akt signaling and upregulation ABC transporters (Gu et al., 2022).

### Tumor suppressive miRNAs and GEM resistance

Multiple tumor suppressive miRNAs also participate in regulating GEM resistance of PC cells through different mechanisms, such as apoptosis, cell proliferation, cell cycle, EMT, CSCs, autophagy and glycolysis. miR-33a, for example, could suppress GEM resistance and cell proliferation in PC via targeting Pim-3 and inhibiting the Akt/GSK-3β/β-catenin signaling pathway (Liang et al., 2015b). Similarly, miR-33a could enhance the sensitivity to GEM in human PC cells through inhibiting β-catenin nuclear translocation, suppressing *survivin*, *cyclin D1* (*CCND1*) and *multi-drug resistance 1* (*MDR-1*) transcription, as well as reducing protein expression of N-cadherin, slug and vimentin (Liang et al., 2015a). miR-497, a downregulated miRNA in GEM-resistant PC cells, could reverse GEM resistance through silencing expression of its target genes *FGF2* and *FGFR1* (Xu et al., 2014). It has been shown that over-expression of miR-210 could also potentiate GEM sensitivity via suppressing expression of its target gene *ABCC5* (Amponsah et al., 2017). In addition, tumor suppressors miR-1285, miR-608, miR-506, miR-30a-5p and miR-146a-5p have been demonstrated to impair GEM resistance of PC by silencing expression of YAP1/EGFR/β-catenin, ribonucleotide reductase M1 (RRM1)/CDA, sphingosine kinase 1 (SPHK1)/Akt/NF-κB, FOXD1/ERK and the tumor necrosis factor receptor-associated factor 6 (TRAF6)/NF-kB p65/P-gp signaling, respectively (Li et al., 2016; Huang et al., 2017; Rajabpour et al., 2017; Zhou et al., 2019; Meng et al., 2020). miR-34 has been found to induce apoptosis, cell cycle arrest in G1 and G2/M phase and sensitize PC cells to GEM via suppressing expression of its target genes *Bcl-2* and *Notch1/2*. Moreover, miR-34 could inhibit growth of CSCs and tumor spheres *in vitro* and tumorigenesis *in vivo* (Ji et al., 2009). Additionally, miR-34 has also been shown to potentiate GEM-induced apoptosis of PC cells through inhibiting expression of *Slug* and elevating expression of *p53 upregulated modulator of apoptosis* (*PUMA*) (Zhang Q A et al., 2018). miR-494, a miRNA with decreased levels in PC tissues and cells, could lead to apoptosis, senescence, G1 phase accumulation and the impaired GEM resistance, through directly silencing the c-myc/sirtuin1(SIRT1) signaling (Liu et al., 2015). In addition, tumor suppressors miR-373-3p, miR-101, miR-101-3p, miR-153 and miR-374b-5p have also been found to reverse GEM resistance in PC via promoting apoptosis (Fan et al., 2016; Hu et al., 2017; Liu et al., 2017; Hu et al., 2018; Sun et al., 2018). It has been found that tumor suppressor miR-410-3p and miR-29c could attenuate the resistance to GEM in PC through inhibiting expression of *High mobility group box 1* (*HMGB1*) and *ubiquitin specific peptidase-22* (*USP22*) and, thus, reducing autophagy, respectively (Xiong et al., 2017; Huang et al., 2018). Similarly, miR-127 could confer GEM sensitivity of PC cells through down-regulating expression of *CCNE1*, *CDKN1A*, *CDKN1B*, *CCND1* and *CDK2*, and promoting cell cycle arrested in S phase (Panebianco et al., 2021).

Tumor suppressive miRNAs are also involved in regulating the EMT process and/or CSC formation which have been associated with development of GEM resistance in PC. For instance, tumor suppressors miR-509-5p and miR-1243 have been found to enhance GEM sensitivity through modulating expression of EMT markers *E-cadherin*, *Z O -1*, *Zinc finger E-box binding homeobox transcription factor 1* (*ZEB1*) and *Snail* in PC (Hiramoto et al., 2017). In addition, miR-200c, a miRNA with significantly reduced levels in human CSCs of PC (PCSCs), could effectively overcome GEM resistance and diminish colony formation of PCSCs (Ma et al., 2015). Similarly, in Capan-1, Capan-2, PANC-1, MIAPaCa-2, BxPC-3 and PL45 PC cell lines, miR-200b levels have been found to be negatively correlated with GEM resistance. Moreover, miR-200b overexpression could enhance GEM sensitivity by modulating expression of EMT markers *ZEB* and *CDH1* (Funamizu et al., 2019). In addition, miR-125a-3p and miR-3656 could also potentiate GEM sensitivity of PC cells through silencing *Fyn* and *RHOF* and interfering EMT process, respectively (Yang S Z et al., 2017; Liu B et al., 2018). The miR-17-92 cluster miRNAs, which were downregulated in chemoresistant PCSCs, could reverses GEM resistance and quiescence through the NODAL/ACTIVIN/TGF-β1 signaling (Cioffi et al., 2015). Moreover, miR-205 has been shown to suppress the PCSCs proliferation and reduce GEM resistance via silencing expression of *tubulin beta 3 class III* (*TUBB3*), *RRM1* and *ZEB1* (Chaudhary et al., 2017). Similarly, tumor suppressor miR-497, downregulated in CSCs from BxPC-3 and ASPC-1 PC cells and PC tissues, has also been found to inhibit GEM resistance and metastasis via directly targeting *NF-κB1*. On the contrary, suppression of miR-497 could dramatically contribute to GEM resistance, migration and invasion of PC CSCs (Yu Q et al., 2022).

Additionally, levels of tumor suppressor miR-142-5p in surgically resected PC tissues have been found to be as a prospective marker to predict GEM response (Ohuchida et al., 2011). Multiple tumor suppressor miRNAs, such as miR-145, miR-429, miR-30a and miR-760, could also reverse GEM resistance of PC through inhibiting expression of *p70S6K1*, *PDCD4*, *SNAI1/IRS1/ERK/AKT, moloney leukemia virus 10 (MOV10) and Integrin β1 (ITGB1),* respectively (Lin et al., 2016; Yu et al., 2017; Wang T et al., 2019; Yang et al., 2019). Interestingly, let-7, miR-211 and miR-20a-5p have also been shown to improve the sensitivity to GEM in PC cells via silencing expression of *RRM2* (Bhutia et al., 2013; Maftouh et al., 2014; Lu et al., 2019). Tumor suppressor miR-7 could reverse GEM resistance of PC cells via modulating poly (ADP-ribose) polymerase 1 (PARP1)/NF-κB axis and cellular senescence (Ye et al., 2021). miR-136-5p has been shown to reduce GEM resistance through silencing expression of *ZNF32* (Xu Y et al., 2020). Tumor suppressor miR-3662 could also reduce GEM resistance and aerobic glycolysis in PDAC cells via suppressing levels of *hypoxia-inducible factor 1ɑ* (*HIF-1ɑ*) (Liu A et al., 2021).

### miRNAs and 5-FU resistance

As a thymidylate synthase inhibitor, 5-FU is commonly used for PC treatments in clinic. 5-FU leads to apoptosis and cell cycle arrest through interfering DNA replication, RNA function and protein synthesis. It has been demonstrated that several oncogenic or tumor suppressor miRNAs contribute to the resistance to 5-FU in PC (Table 3).

**TABLE 3:** miRNAs modulating 5-FU resistance in pancreatic cancer.

miRNAs	Expression^a^	Genes and pathways	References
miR-21	upregulated	PTEN/PDCD4	Wei et al. (2016)
		-	Zhao et al. (2015)
		-	Hwang et al. (2010)
miR-221	upregulated	-	Zhao et al. (2015)
miR-221-3p	upregulated	RB1	Zhao et al. (2016)
miR-296-5p	upregulated	BOK/vimentin/N-cadherin	Okazaki et al. (2020)
miR-320a	upregulated	PDCD4	Wang et al. (2016)
miR-499a-5p	upregulated	PI3K/Akt, PTEN, P-gp,MRP1, BCRP	Ouyang et al. (2021)
miR-137	downregulated	PTN	Xiao et al. (2014)
miR-138-5p	downregulated	vimentin	Yu et al. (2015)
miR-494	downregulated	SIRT1/c-myc	Liu et al. (2015)

^a^ miRNAs, either upregulated or downregulated in 5-FU, resistant pancreatic cancer cells. This table shows 9 miRNAs, whose expression levels and potential targets in 5-FU, resistance of pancreatic cancer.

Multiple oncogenic miRNAs, such as miR-21, miR-221, miR-296-5p, miR-320a and miR-499a-5p, have been found to be involved in 5-FU resistance in PC. For example, miR-21 could confer 5-FU resistance in human PATU8988 and PANC-1 PC cells via inhibiting the expression of tumor suppressor genes *PTEN* and *PDCD4* (Wei et al., 2016). In tumor-initiating stem-like PC cells (L3.6 pL), suppression of miR-21 and miR-221 have been shown to reduce side population (SP) cell fraction and reverse 5-FU resistance (Zhao et al., 2015). Moreover, low expression of miR-21 not only was associated with good prognosis of PDAC cases treated with 5-FU-based adjuvant regimens in two independent cohorts, but also could potentiate the sensitivity to 5-FU in PL45 and HPAF-II PC cells (Hwang et al., 2010). miR-221-3p could promote cell proliferation, EMT and 5-FU resistance via targeting the *RB1* 3′-UTR region in PC (Zhao et al., 2016). In addition, oncogenic miR-296-5p and miR-320a contribute to 5-FU resistance of PC cells by modulating *BOK*, *vimentin*, *N-cadherin* and *PDCD4* expression levels, respectively (Wang et al., 2016; Okazaki et al., 2020). miR-499a-5p could promote cell proliferation, migration and 5-FU resistance in PC cells through targeting *PTEN* and activating the PI3K/Akt pathway. Moreover, miR-499a-5p has been shown to influence the expression of MDR-related genes, including *adenosine triphosphate* (*ATP*) *binding cassette subfamily B member 1* (*P-gp*), *ATP binding cassette subfamily C member 1* (*MRP1*), and *ATP binding cassette subfamily G member 2* (*BCRP*) (Ouyang et al., 2021).

By contrast, several tumor suppressor miRNAs can reverse 5-FU resistance of PC. MiR-137, for instance, has been found to be markedly downregulated in PC cell lines and tissues. Over-expression of miR-137 could sensitize cells to 5-FU through inhibiting *pleiotropic growth factor* (*PTN*) expression (Xiao et al., 2014). In addition, miR-138-5p and miR-494, which were both downregulated in PC tissues and cell lines, have been shown to increase 5-FU sensitivity through targeting *vimentin*, *SIRT1* and *c-myc* expression, respectively (Liu et al., 2015; Yu et al., 2015).

### miRNAs and resistance to other drugs

ADM, DDP, oxaliplatin and FOLFIRINOX (a combination regimen of folinicacid, 5-FU, irinotecan and oxaliplatin), targeted therapy drugs and immunotherapy agents are also used during PC clinical treatments. It has been found that several tumor suppressor miRNAs, including miR-137, miR-142 and miR-212, could weaken ADM resistance of PC. For example, through targeting *ATG5* and improving autophagy, exogenous expression of miR-137 could enhance ADM sensitivity and promote apoptosis in PC cells (Wang Z C et al., 2019). Interestingly, the plectin-1(PL-1)/miR-212 nanoparticles could significantly promote ADM-induced apoptosis and autophagy by silencing the expression of *ubiquitin specific peptidase 9 X-linked* (*USP9X*) in PC cells (Chen Y et al., 2019). Oncogenic miR-223 has been shown to promote proliferation and DDP resistance via targeting *forkhead transcription factor O subfamily 3a* (*FoxO3a*) in PC cells (Huang et al., 2019). In human MiaPaCa2 and BxPC3 PC cells harboring *P53* mutations, exogenous expression of tumor suppressor miR-34 could not only result in cell cycle arrest, apoptosis, the reduced tumor-initiating cell population and tumor sphere growth, but also sensitize the cells to DDP through down-regulating *Notch1/2* and *Bcl-2* expression (Ji et al., 2009). miR-100, a downregulated miRNA in PC tissues and cell lines, could increase DDP sensitivity and suppress tumor growth *in vivo* via targeting *fibroblast growth factor receptor 3* (*FGFR3*) (Li et al., 2014). In addition, it has been found that miR-374b, a downregulated miRNA in DDP-resistant PC cell line BxPC3-R, contributed to the acquired DDP resistance, at least partly by targeting *ATP7A* (*ATPase, Cu* ^*2+*^ *Transporting, Alpha Polypeptide*) and *clusterin* (*CLU*) (Schreiber et al., 2016). Tumor suppressor miR-1291-5p could act as a metabolism regulator and potentiate the sensitivity to DDP via diminishing *glucose transporter protein type 1* (*GLUT1*) expression and GLUT1-mediated glycolysis in ASPC-1 and PANC-1 PC cells (Tu et al., 2020). Laura and colleagues showed that inhibition of miR-181a-5p could potentiate oxaliplatin sensitivity of PC cells via suppressing expression of *ATM*. Moreover, PC patients with better response to FOLFIRINOX displayed lower levels of miR-181a-5p both in cancerous tissues and plasma specimens (Meijer et al., 2020). Interestingly, through regulation of DNA damage, miR-1307 has been shown to modulate FOLFIRINOX sensitivity in PDAC cells (Carotenuto et al., 2021).

In addition to be involved in chemoresistance, a few miRNAs have been shown to confer resistance to targeted therapy and immunotherapy in PC. For instance, silencing oncogeneic miR-21 could potentiate the sensitivity to sunitinib in PDAC (Passadouro et al., 2014). Izumchenko and colleagues showed that silencing of tumor suppressor miR-200 could upregulate the expression of negative EGFR regulator of *mitogen-inducible gene 6* (*MIG6*) in the process of transforming growth factor β (TGFβ)-mediated EMT. Moreover, the ratio of *MIG6* mRNA to miR-200 (*MIG6* mRNA/miR-200) was negatively correlated with erlotinib response not only in cancer cell lines with diverse tissue origins *in vitro*, but also in xenografts derived from PC patients carrying wild-type EGFR *in vivo* (Izumchenko et al., 2014). Similarly, tumor suppressor miR-497 could impact erlotinib resistance via modulating expression levels of *fibroblast growth factor 2* (*FGF2*) and *fibroblast growth factor receptor 1* (*FGFR1*) (Xu et al., 2014). Through inhibiting the expression of *erythropoietin-producing hepatocellular receptor 2* (*EphA2*), tumor suppressor miR-124 has also been found to improve erlotinib sensitivity in Capan-1 PC cells with *K-RAS* mutations (Du et al., 2019). Table 4.

**TABLE 4:** miRNAs modulating resistance to other drugs in pancreatic cancer.

miRNAs	Expression^a^	Genes and pathways	Drugs	References
miR-137	downregulated	ATG5	ADM	Wang Z C et al. (2019)
miR-212	downregulated	USP9X	ADM	Chen Y et al. (2019)
miR-223	upregulated	FoxO3a	DDP	Huang et al. (2019)
miR-34	downregulated	Bcl-2/Notch1/Notch2	DDP	Ji et al. (2009)
miR-100	downregulated	FGFR3	DDP	Li et al. (2014)
miR-374b	downregulated	ATP7A/CLU	DDP	Schreiber et al. (2016)
miR-1291-5p	downregulated	GLUT1	DDP	Tu et al. (2020)
miR-181a-5p	upregulated	ATM	Oxaliplatin, FOLFIRINOX	Meijer et al. (2020)
miR-1307	upregulated	CLIC5	FOLFIRINOX	Carotenuto et al. (2021)
miR-21	upregulated	-	Sunitinib	Passadouro et al. (2014)
miR-200	downregulated	MIG6	Erlotinib	Izumchenko et al. (2014)
miR-497	downregulated	FGF2/FGFR1	Erlotinib	Lin et al. (2020)
miR-124	downregulated	EphA2	Erlotinib	Du et al. (2019)

^a^ miRNAs, either upregulated or downregulated in other drugs resistant pancreatic cancer cells. This table shows 12 miRNAs, whose expression levels and potential targets in other drugs resistance of pancreatic cancer.

## lncRNAs and drug resistance

LncRNAs could be divided into four types according to their location in the genome: intronic lncRNAs, intergenic lncRNAs, divergent lncRNAs and antisense lncRNAs (Lee, 2012). Accumulating evidences demonstrated that multiple lncRNAs, functioning as oncogenes or tumor suppressors, contribute to tumorigenesis, disease progression and therapy response by regulating specific target genes or signaling pathways (Johnsson et al., 1991; Feng et al., 2020; Gong et al., 2020; He et al., 2020; Zhu et al., 2020; Qu et al., 2021). In PC, lncRNAs have been found to be involved in development of drug resistance (Table 5; Table 6).

**TABLE 5:** LncRNAs modulating gemcitabine resistance in pancreatic cancer.

LncRNAs	Expression^a^	Genes and pathways	References
HOTTIP	upregulated	HOXA13	Li et al. (2015)
GSTM3TV2	upregulated	let-7/LAT2/OLR1	Xiong et al. (2019)
MALAT-1	upregulated	Sox2	Jiao et al. (2015)
PVT1	upregulated	-	You et al. (2011)
		miR-619-5p/Pygo2/ATG14	Zhou et al. (2020)
		miR-143/HIF-1α/VMP1	Liu Y F et al. (2021)
TUG1	upregulated	ERK	Yang et al. (2018)
DGCR5	upregulated	miR-3163/TOP2A,Wnt/β-catenin	Liu S L et al. (2021)
HIF1A-AS1	upregulated	AKT/YB1/HIF-1α	Xu F et al. (2021a)
UCA1	upregulated	SOCS3/EZH2	Chi et al. (2021)
SNHG16	upregulated	Smad4	Yu Q et al. (2022)
SH3BP5-AS1	upregulated	miR-139-5p/Wnt/CTBP1	Lin et al. (2022)
LINC00460	upregulated	PDAP1/PDGFA/PDGFR	Zhu et al. (2022)
linc-DYNC2H1-4	upregulated	miR-145/ZEB1/Lin28/Nanog/Sox2/Oct4	Gao et al. (2017)
SLC7A11-AS1	upregulated	NRF2, ROS	Yang Q et al. (2020)
SBF2-AS1	upregulated	miR-142-3p/TWF1	Hua et al. (2019)
HCP5	upregulated	miR-214-3p/HDGF	Liu et al. (2019)
SNHG14	upregulated	miR-101/RAB5A/ATG4D	Zhang et al. (2019)
LINC01559	upregulated	P62, LC3, caspase3, PARP	Deng et al. (2020)
LINC00346	upregulated	miR-188-3p/BRD4	Shi et al. (2019)
SNHG8	upregulated	caspase-3/PARP	Song et al. (2018)
HOST2	upregulated	apoptosis	An and Cheng (2020)
ANRIL	upregulated	miR-181a, HMGB1	Wang et al. (2021)
NEAT1	upregulated	miR-491-5p/Snail/SOCS3	Wu et al. (2023)
GAS5	downregulated	miR-221/SOCS3	Liu B et al. (2018)
		miR-181c-5p/Hippo	Gao et al. (2018)
AB209630	downregulated	PI3K/Akt	Wang et al. (2018)
MEG3	downregulated	snail	Ma T et al. (2018)
DLEU2L	downregulated	miR-210-3p, BRCA2	Xu F et al. (2021b)
DBH-AS1	downregulated	miR-3163/USP44	Ye et al. (2022)
DSCR9	downregulated	miR-21-5p/BTG2	Huang et al. (2022)

^a^ lncRNAs, either upregulated or downregulated in gemcitabine resistant pancreatic cancer cells. This table shows 28 lncRNAs, whose expression levels and potential targets in gemcitabine resistance of pancreatic cancer.

**TABLE 6:** LncRNAs modulating resistance to other drugs in pancreatic cancer.

LncRNAs	Expression^a^	Genes and pathways	Drugs	References
TUG1	upregulated	miR-376b-3p/DPD	5-FU	Tasaki et al. (2021)
HOTTIP	upregulated	miR-137	DDP	Yin et al. (2020)
UPK1A-AS1	upregulated	IL8, Ku70, Ku80	Oxaliplatin	Zhang et al. (2022)
HOTAIR	upregulated	EZH2/DR5	TRAIL	Yang R M et al. (2017)
SNHG7	upregulated	Notch1/Jagged1/Hes-1, MSCs	FOLFIRINOX	Cheng et al. (2021)
LINC02432	upregulated	miR-98-5p/HK2	EGFR, MEK and ERK inhibitors	Tan et al. (2022)
GAS5	downregulated	miR-181c-5p/Hippo	5-FU	Gao et al. (2018)

^a^ lncRNAs, either upregulated or downregulated in other drugs resistant pancreatic cancer cells. This table shows 7 lncRNAs, whose expression levels and potential targets in other drugs resistance of pancreatic cancer.

### lncRNAs and GEM resistance

Similar with miRNAs, several lncRNAs have been shown to participate in GEM resistance in PC, including oncogenic lncRNAs and tumor suppressive lncRNAs molecules (Table 5).

### Oncogenic lncRNAs and GEM resistance

Multiple oncogenic lncRNAs have been shown to contribute to GEM resistance in PC, such as *HOXA transcript at the distal tip* (*HOTTIP*), *glutathione S-transferase mu 3, transcript variant 2* (*GSTM3TV2*), *metastasis-associated lung adenocarcinoma transcript 1* (*MALAT1*), *plasmacytoma variant translocation 1* (*PVT1*) and *DiGeorge syndrome critical region gene 5* (*DGCR5*).

HOTTIP, an overexpressed lncRNA in PDAC tissues and cells, has been found to promote GEM resistance by modulating levels of *HOXA13* in PDAC cells. In contrast, silencing HOTTIP could lead to enhanced sensitivity of PC cells to GEM (Li et al., 2015). LncRNA GSTM3TV2, which was significantly upregulated in GEM-resistant PC cells, could confer GEM resistance by competitively sponging let-7 and subsequently up-regulating expression of *L-type amino acid transporter 2* (*LAT2*) and *oxidized low-density lipoprotein receptor 1* (*OLR1*). Moreover, the increased levels of GSTM3TV2 in PC tissues have been significantly associated with worse prognosis, indicating that GSTM3TV2 may be a novel prognostic marker and therapeutic target in PC (Xiong et al., 2019). LncRNA MALAT-1, initially identified as a prognostic marker for lung cancer patients, was involved in PC chemoresistance. It has been demonstrated that MALAT-1 could not only reduce GEM sensitivity, but also potentiate the proportion and self-renewal ability of PCSCs, by up-regulating expression of self-renewal related factor *Sox2* (Jiao et al., 2015). Interestingly, over-expression of lncRNA PVT1 could promote GEM resistance in naïve ASPC-1 PC cells (You et al., 2011). Moreover, PVT1 could confer the resistance to GEM in PC via regulating the miR-619-5p/Pygo2 and miR-619-5p/ATG14 axes, which lead to dysregulated autophagic activities and the Wnt/β-catenin signaling (Zhou et al., 2020). Similarly, silencing of PVT1 could suppress autophagy and promote GEM sensitivity through modulating the miR-143/HIF-1α/VMP1 axis in PC (Liu S L et al., 2021). In addition, oncogenic lncRNA TUG1 has also been shown to contribute to GEM resistance of PDAC cells via inducing expression of SCH772984 which is an ERK pathway suppressor (Yang et al., 2018). LncRNA *DiGeorge syndrome critical region gene 5* (*DGCR5*) could enhance GEM resistance via functioning as a ceRNA through sponging miR-3163 to inhibit the Wnt/β-catenin signaling and modulate expression of *DNA topoisomerase 2-alpha* (*TOP2A*) (Liu Y F et al., 2021). LncRNA *HIF1A-AS1* (*antisense RNA1 of HIF-1α*) has been found to prevent GEM sensitivity of PC cells through activating the AKT/YB1/HIF-1α signaling and promoting glycolysis (Xu F et al., 2021a). Interestingly, lncRNA u*rothelial carcinoma-associated 1* (*UCA1*), which was delivered by hypoxic pancreatic stellate cells (PSCs)-derived exosomes (HPSC-EXO), has also been shown to enhance GEM resistance in PC (Chi et al., 2021). Through enhancer of zeste homolog 2 (EZH2) -mediated epigenetic modification, lncRNA *small nucleolar RNA host gene 16 (SNHG16)* has been found to confer GEM resistance via diminishing *SMAD family member (Smad4)* expression in PC (Yu Y et al., 2022). Via sponging miR-139-5p and activating Wnt pathway, and subsequently increasing the expression of *ezh2,* lncRNA *SH3BP5-AS1* has been demonstrated to contribute to GEM resistance of PC cells (Lin et al., 2022).

Accumulating evidences demonstrated that CAFs are critically involved in chemoresistance (Qin et al., 2019; Zhang H et al., 2020; Yang et al., 2022). LINC00460, a lncRNA molecule mainly located in the cytoplasm, has been shown not only to correlate with GEM response in PDAC patients, but also to regulate GEM resistance of CAFs through mediating the cellular communication of PDAC cancer cells and CAFs by platelet derived growth factor subunit A (PDGFA) associated protein 1 (PDAP1)/PDGFA/PDGFR signaling pathway (Zhu et al., 2022).

Additionally, EMT and CSCs have also been shown to promote lncRNAs-mediated chemotherapy resistance. Linc-DYNC2H1-4, an upregulated intergenic lncRNA in GEM-resistant BxPC-3-GEM PC cells, could promote EMT and stemness of the parental sensitive cells. In cells, Linc-DYNC2H1-4 could sponge miR-145 and elevate levels of several EMT key players including *ZEB1* and CSC markers including *Lin28*, *Nanog*, *Sox2* and *Oct4* (Gao et al., 2017). LncRNA *SLC7A11-AS1*, which was over-expressed in PDAC tissues and GEM-resistant cell lines, has also been found to potentiate PDAC stemness and GEM resistance through stabilizing nuclear factor erythroid-2-related factor 2 (NRF2) and stimulating intracellular reactive oxygen species (ROS) (Yang Z et al., 2020). In addition, oncogenic lncRNA *SBF2-AS1* could promote EMT and GEM resistance in PC via sponging miR-142-3p and up-regulating expression of *twinfilin 1* (*TWF1*) (Hua et al., 2019).

Autophagy also contributes to drug resistance and cancer progression (Kim and Lee, 2014; Levy et al., 2017; Wu and Zhang, 2020). It has been found that lncRNAs *HLA complex P5* (*HCP5*) and *SNHG14* could confer GEM resistance of PC through sponging miR-214-3p and miR-101 and activating autophagy (Liu et al., 2019; Zhang et al., 2019). LncRNA LINC01559 has also been found to confer GEM resistance by promoting autophagy and inhibiting apoptosis (Deng et al., 2020). In addition, oncogenic lncRNAs LINC00346, small nucleolar RNA host gene 8 (SNHG8) and HOST2, have also been shown to confer GEM resistance of PC via regulating the miR-188-3p/BRD4 axis, the caspase-3/PARP axis and apoptosis (Song et al., 2018; Shi et al., 2019; An and Cheng, 2020). Additionally, lncRNA ANRIL could enhance GEM resistance of PC cells through inhibiting miR-181a expression and modulating autophagy induced by HMGB1 (Wang et al., 2021).

Mesenchymal stem cells (MSCs), a crucial cell type in tumor micro-environment, may contribute to drug resistance in multiple neoplasms via producing protective cytokines or influencing gene expression (Houthuijzen et al., 2012; Liu C et al., 2021). It has been found that extracellular vesicle -loaded oncogenic lncRNA nuclear paraspeckle assembly transcript 1 (NEAT1) from adipose-derived MSCs could promote GEM resistance in PC by regulating the miR-491-5p/snail/suppressor of cytokine signaling 3 (SOCS3) signaling pathway (Wu et al., 2023).

### Tumor suppressor lncRNAs and GEM resistance

Several tumor suppressor lncRNAs have also been found to be associated with the resistance to therapeutic agents in PC. For instance, lncRNA *growth arrest-specific 5* (*GAS5*) has been demonstrated to suppress GEM resistance of PC cells through inhibiting miR-221 expression and increasing *SOCS3* expression (Liu G et al., 2018). Interestingly, GAS5 has also been found to antagonize GEM resistance in PC cells via negatively regulating miR-181c-5p expression and subsequently activating the Hippo signaling (Gao et al., 2018). AB209630, an evidently downregulated lncRNA in PDAC tissues, could improve GEM sensitivity of PDAC cells by modulating the PI3K/Akt pathway. Moreover, high levels of lncRNA *AB209630* were associated with good prognosis of PDAC patients (Wang et al., 2018). MEG3, which is a downregulated lncRNA in PC tissues and cells, has been found to sensitize GEM in PC cells. On the contrary, silencing MEG3 led to inhibited GEM sensitivities. Moreover, low expression of MEG3 in PC patients were associated with GEM resistance and poor outcomes (Ma L et al., 2018). LncRNA DLEU2L (deleted in lymphocytic leukemia 2-like), which was downregulated in PC tissues, has been shown to lessen GEM resistance of PC cells via modulating expression of *BRCA2* (Xu F et al., 2021b). Through modulating the miR-3163/USP44 axis in PC cells, tumor suppressive lncRNA DBH-AS1 has been found to reverse GEM resistance and inhibit cell growth (Ye et al., 2022). Most recently, down syndrome critical region 9 (DSCR9), a downregulated lncRNA in PC cells and tissues, has also been shown to suppress the proliferation, invasion and GEM resistance by miR-21-5p/BTG anti-proliferation factor 2 (BTG2) axis (Huang et al., 2022).

### lncRNAs and resistance to other drugs

In addition to participate in GEM resistance, lncRNAs have also been shown to promote other drugs resistance, such as 5-FU, DDP, oxaliplatin, FOLFIRINOX and targeted therapy agents (Table 6).

### Oncogenic lncRNAs and resistance to other drugs

LncRNA TUG1 could promote 5-FU resistance in PC cells via inhibiting expression of miR-376b-3p and elevating *dihydropyrimidine dehydrogenas*e (*DPD*) expression (Tasaki et al., 2021). HOTTIP has been found to promote DDP resistance of PC cells through silencing miR-137 (Yin et al., 2020). Similarly, oncogenic lncRNA UPK1A-AS1 could confer oxaliplatin resistance induced by paracrine IL8 derived from CAFs via activating Ku70 and Ku80 interaction and, thus, promoting nonhomologous end joining (NHEJ) and DNA double-strand break (DSB) repair. On the contrary, silencing UPK1A-AS1 could reverse oxaliplatin resistance of PC cells (Zhang et al., 2022). Interestingly, oncogenic lncRNA *HOX transcript antisense gene RNA* (*HOTAIR*) has been demonstrated to confer resistance to TNF-related apoptosis-inducing ligand (TRAIL) via interacting with the epigenetic regulator EZH2 and inhibiting *TRAIL receptor death receptor 5* (*DR5*) expression. Silencing HOTAIR could enhance TRAIL-induced apoptosis in TRAIL-resistant PC cells. On the contrary, HOTAIR over-expression could inhibit apoptosis induced by TRAIL in TRAIL-sensitive cells, indicating that HOTAIR may act as a potential therapeutic target to conquer TRAIL-resistance in PC (Yang R M et al., 2017). In addition, it has been shown that lncRNA small nucleolar RNA host gene 7 (SNHG7) could modulate activities of MSCs and confer FOLFIRINOX resistance in PC through the Notch1/Jagged1/Hes-1 signaling pathway (Cheng et al., 2021). Aerobic glycolysis, a hallmark of PC, has been shown to promote tumorigenesis and progression via multiple mechanisms. Glycolysis-related lncRNA LINC02432 could inhibit ferroptosis and predict drug sensitivity to EGFR inhibitors (afatinib and sapitinib), MEK inhibitors (trametinib, PD0325901 and selumetinib) and ERK inhibitors (VX-11e, Ulicocitinib, SCH772984, and ERK_6604) in PC by regulating miR-98-5p/hexokinase 2 (HK2) axis (Tan et al., 2022).

### Tumor suppressive lncRNAs and resistance to other drugs

Unlike tumor suppressive miRNA, few tumor suppressor lncRNA has been found to be associated with other drugs resistance. Until now, only tumor suppressive lncRNA GAS5 could prevent 5-FU resistance in PC cells by sponging miR-181c-5p and subsequently activating the Hippo signaling (Gao et al., 2018).

## circRNAs and drug resistance

CircRNAs, a special class of ncRNAs with covalently closed-loop structure, have been shown to function as important regulator in various tumors, including PC (Kristensen et al., 2019; Wong et al., 2020). Amounting data indicated that circRNAs function in carcinogenesis and progression of PC (Shao et al., 2018; Chen W et al., 2019). Importantly, several circRNAs have been associated with development of drug resistance in PC (Table 7).

**TABLE 7:** CircRNAs modulating drug resistance in pancreatic cancer.

CircRNAs	Expression^a^	Genes and pathways	Drugs	References
circHIPK3	upregulated	miR-330-5p, RASSF1	GEM	Liu et al. (2020)
circZNF91	upregulated	miR-23b-3p	GEM	Zeng et al. (2021)
circ-MTHFD1L	upregulated	miR-615-3p/RPN6	GEM	Chen et al. (2022)
circ_0074298	upregulated	miR-519, SMOC	GEM	Hong et al. (2022)
circFARP1	upregulated	LIF/STAT3	GEM	Hu et al. (2022)
circLMTK2	upregulated	miR-485-5p/PAK1	GEM	Lu et al. (2022)
circ_0092367	downregulated	miR-1206/ESRP1	GEM	Yu et al. (2021)
circ_0013587	downregulated	miR-1227/E-cadherin	Erlotinib	Xu H et al. (2021)

^a^ circRNAs, either upregulated or downregulated in resistant pancreatic cancer cells. This table shows 8 circRNAs, whose expression levels and potential targets in drugs resistance of pancreatic cancer.

### circRNAs and GEM resistance

Through circRNA-sequencing analyses of GEM-resistant PANC-1-GR PC cells and wide-type PANC-1 cells, it has been found that 68 upregulated circRNAs and 58 downregulated ones in PANC-1-GR cells. Seven upregulated circRNAs (hsa_circ_0000522, hsa_circ_0000943, chr4:174305802-174325101+, chr1:169947226-170001116-, chr14:101402109-101464448+, chr4:52729603-52780244+, chr6:29901995-29911250+) and three downregulated circRNAs (hsa_circ_0070033, hsa_circ_0008161, hsa_circ_0006355) were successfully verified by the qRT-PCR assay. Among which, chr14:101402109-101464448 + and chr4:52729603-52780244+ have been found to be highly expressed in plasma of PC patients, who showed no response to GEM treatment. Silencing these two circRNAs could restore GEM sensitivity of PANC-1-GR cells (Shao et al., 2018). Similarly, Xu et al. found that the top 10 upregulated circRNAs in SW1990/GZ PC cells were circ_101672, circ_004077, circ_003251, circ_102,402, circ_074298, circ_089762, circ_003596, circ_089761, circ_002178 and circ_102,403. In contrast, the top 10 downregulated circRNAs were circRNA_101,543, circRNA_102,747, circRNA_000926, circRNA_059665, circRNA_103827, circRNA_406521, circRNA_103128, circRNA_104490, circRNA_103829 and circRNA_070037 (Xu et al., 2018).

CircHIPK3, an upregulated circRNA in PC tissues and GEM-resistant PC cells, has also been found to confer GEM resistance through regulating *RASSF1* expression (Liu et al., 2020). Through depriving the suppression of miR-23b-3p on expression level of deacetylase SIRT1 and leading to increased glycolysis, circZNF91 has been shown to confer GEM resistance in PC cells (Zeng et al., 2021)**.** Oncogenic circ-MTHFD1L could induce DNA damage repair and confer GEM resistance in PDAC through the miR-615-3p/RPN6 axis. On the contrary, inhibition of circ-MTHFD1L combined with olaparib could reverse GEM resistance (Chen et al., 2022). Similarly, circ_0074298 has also been found to promote GEM resistance and PC progression through sponging miR-519 and regulating *SMOC* expression (Hong et al., 2022). Interestingly, circFARP1 could enable CAFs to promote GEM resistance in PC via the leukemia inhibitory factor (LIF)/STAT3 axis (Hu et al., 2022). CircLMTK2, an over-expressed circRNA in GEM-resistant PC cells and PC tissues, could contribute to GEM resistance via modulating p21 (RAC1) activated kinase 1 (PAK1) by sponging miR-485-5p (Lu et al., 2022).

Similarly, tumor suppressive circRNA has also been demonstrated to participate in the resistance to GEM. For example, tumor suppressor circ_0092367 could inhibit EMT and enhance GEM sensitivity in PC cells through regulating the miR-1206/epithelial splicing regulatory protein 1 (ESRP1) axis (Yu et al., 2021).

### circRNAs and resistance to other drugs

To date, no report has shown that oncogenic circRNAs could involve in the resistance to other drugs. In the aspect of tumor suppressor circRNAs, only circ_0013587 has been found to reverse erlotinib resistance through modulating the miR-1227/E-cadherin signaling in PC cells (Xu H et al., 2021).

## Conclusion

**FIGURE 1:**
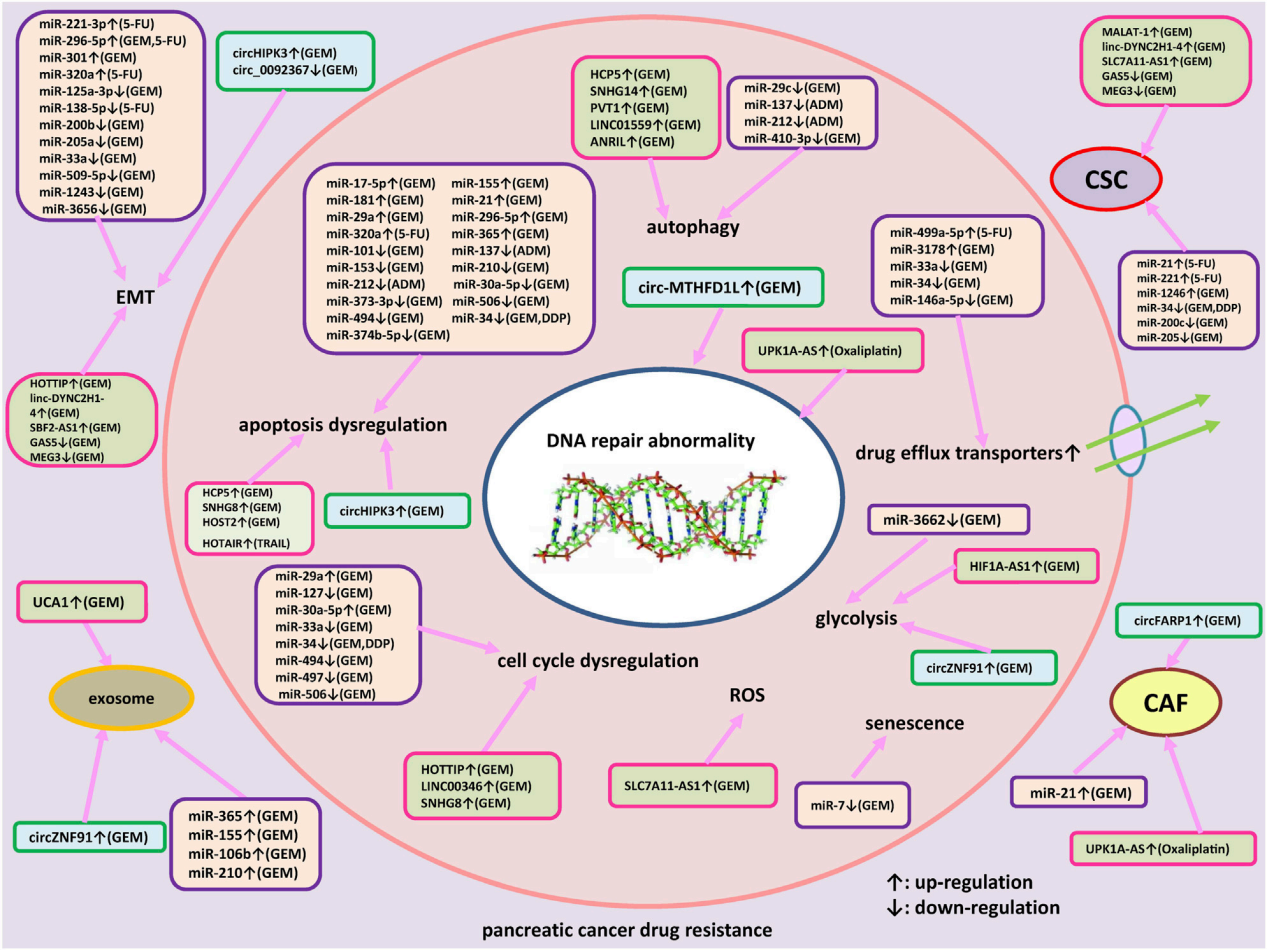
An outlined diagram of miRNAs, lncRNAs and circRNAs participated in the resistance to therapeutic agents in pancreatic cancer. Several miRNAs, lncRNAs, and circRNAs have been shown to be associated with pancreatic cancer drug resistance via regulating apoptosis, cell cycle arrest, DNA damage repair, cell proliferation, autophagy, epithelial-mesenchymal transition (EMT), drug efflux transporter, reactive oxygen species, glycolysis and cancer stem cells (CSC) through regulating specific signaling pathways and target genes.

Many ncRNAs have been shown to be involved in the pathogenesis and progression of PC, indicating the potential roles of ncRNAs as biomarkers for early diagnosis, as promising prognostic and predictive markers for the identification of candidates amenable to adjuvant treatment, enabling a personalized clinical approach. Interestingly, a number of studies attempted to investigate the diagnostic value of circulating miRNAs in PC (Previdi et al., 2017). Recently, a pilot study has proposed that an exosomal four miRNA biomarker panel, consisting of miR-93-5p, miR-339-3p, miR-425-5p, and miR-425-3p, may provide a promising avenue for PC screening (Makler and Asghar, 2023). Although the diagnostic and prognostic potential of ncRNAs have shown the promising results, large and prospective validation studies should be implemented before they can enter clinical practice. Also, ncRNAs can be used as targets for novel therapeutics of PC patients. Accumulating evidences indicated that different kinds of ncRNAs have been reported to be involved in drug resistance of PC. As shown in Figure 1, we summarized how miRNAs, lncRNAs and circRNAs contribute to resistance development to therapeutic agents and the underlying molecular mechanisms in PC. Interestingly, drug resistance may be reversed through targeting specific endogenous miRNAs, lncRNAs and/or circRNAs. Several approaches could be employed to inhibit expression of dysregulated ncRNAs, such as small interfering RNAs (siRNAs), short hairpin RNAs (shRNAs), antagomirs, anti-oligonucleotides (ASOs), clustered regulatory interspaced short palindromic repeats-associated endonuclease 9 (CRISPR/Cas9)-based genome editing, small molecule inhibitors of ncRNAs, and artificial miRNA sponges (Lin et al., 2020; Pandya et al., 2020). The strategies which could restore the normal levels of tumor suppressor ncRNAs involved in drug resistance of PC include to replace or substitute these ncRNAs through synthetic ncRNA-like molecules, for example, miRNA mimics. However, multiple challenges for therapeutic targeting ncRNAs remain to be faced, such as off-target effects, lack of efficient carrier systems, immune related toxicities, tolerability and other side effects. Progresses in ncRNAs delivery vector systems help to increase the potential for ncRNA-based treatment. Due to the improved circulation time and diminished recognition by the immune system of host, nanoparticles have been shown to function as efficient vectors for various gene therapies in PC patients, including siRNAs and miRNAs (Kurtanich et al., 2019). Through searching the database of http://clinicaltrials.gov, no clinical trials based on ncRNAs therapeutics in PC were found currently. The combined therapeutic modalities based on the manipulation of ncRNAs’ levels and traditional treatments, i.e. chemotherapy, molecular targeted therapy or immunotherapy, may be a promising approach to overcome drug resistance and help to improve prognosis of advanced PC patients. Nevertheless, a serious issue is how to select the appropriate target molecules from a huge number of ncRNA candidates. More importantly, clinical studies and translational studies are also needed for conquering drug resistance in PC.

## Figures and Tables

**Table:** 

**PC**	Pancreatic cancer
**PDAC**	Pancreatic ductal adenocarcinoma
**GEM**	Gemcitabine
**ADM**	Adriamycin
**DDP**	Cisplatin
**5-FU**	5-fluorouracil
**TKI**	Tyrosine kinase inhibitor
**EGFR**	Epidermal growth factor receptor
**mTOR**	Mammalian target of rapamycin
**CTLA-4**	Cytotoxic T lymphocyte antigen-4
**PD-1**	Programmed cell death protein 1
**EMT**	Epithelial-mesenchymal transition
**CSCs**	Cancer stem cells
**ncRNAs**	Noncoding RNA
**lncRNA**	Long noncoding RNA
**Nt**	Nucleotide
**sncRNA**	Short ncRNAs
**miRNA/miR**	MicroRNA
**circRNA**	Circular RNA
**snoRNA**	Small nucleolar RNA
**piRNA**	PIWI interacting RNA
**MIG6**	Mitogen-inducible gene 6
**TGF**	Transforming growth factor
**FGF2**	Fibroblast growth factor 2
**FGFR1**	Fibroblast growth factor receptor 1
**EphA2**	Erythropoietin-producing hepatocellular receptor 2
**3′-UTR**	3′-Untranslated region
**Dkk1**	Dikkopf-1
**sFRP2**	Secreted frizzled related protein 2
**PTEN**	Phosphatase and tensin homologue
**PDCD4**	Programmed cell death 4
**CAFs**	Cancer-associated fibroblasts
**PDGF**	Platelet-derived growth factor
**MMP-3**	Matrix metalloproteinase-3
**CCL7**	Chemokine (C-C motif) ligand 7
**SHC1**	Src Homology 2 Domain Containing 1
**MDE**	Macrophage-derived exosomes
**CYLD**	Cylindromatosis
**DCK**	Deoxycytidine kinase
**CDH1**	Cadherin 1
**BOK**	Bcl2-related ovarian killer
**TFAP2C**	Transcription factor activating protein 2 gamma
**KLF6**	Krüppel-like factor 6
**SNF**	Sucrose nonfermentable
**MDR-1**	Multi-drug resistance 1
**RRM1**	Ribonucleotide reductase M1
**SPHK1**	Sphingosine kinase 1
**TRAF6**	Tumor necrosis factor receptor-associated factor 6
**PUMA**	P53 upregulated modulator of apoptosis
**HMGB1**	High mobility group box 1
**USP**	Ubiquitin specific peptidase
**CCND1**	Cyclin D1
**ZEB1**	Zinc finger E-box binding homeobox transcription factor 1
**PCSCs**	CSCs of PC
**TUBB3**	Tubulin beta 3 class III
**MOV10**	Moloney leukemia virus 10
**ITGB1**	Integrin β1
**PARP1**	Poly (ADP-ribose) polymerase 1
**HIF-1ɑ**	Hypoxia-inducible factor 1ɑ
**DNA-PKcs**	DNA-dependent protein kinase catalytic subunit
**SP**	Side population
**PL-1**	Plectin-1
**USP9X**	Ubiquitin specific peptidase 9, X-linked
**FoxO3a**	Forkhead transcription factor O subfamily 3a
**FGFR3**	Fibroblast growth factor receptor 3
**ATP7A**	ATPase, Cu++ Transporting, Alpha Polypeptide
**CLU**	Clusterin
**GLUT1**	Glucose transporter protein type 1
**HOTTIP**	HOXA transcript at the distal tip
**GSTM3TV2**	Glutathione S-transferase mu 3, transcript variant 2
**MALAT1**	Metastasis-associated lung adenocarcinoma transcript 1
**PVT1**	Plasmacytoma variant translocation 1
**HOTAIR**	HOX transcript antisense gene RNA
**LAT2**	L-type amino acid transporter 2
**OLR1**	Oxidized low-density lipoprotein receptor 1
**TOP2A**	Topoisomerase 2-alpha
**DGCR5**	DiGeorge syndrome critical region gene 5
**UCA1**	Urothelial carcinoma-associated 1
**PSC**	Pancreatic stellate cell
**HPSC-EXO**	Hypoxic pancreatic stellate cells derived exosome
**NHEJ**	Nonhomologous end joining
**DSB**	DNA double-strand break
**ROS**	Reactive oxygen species
**NRF2**	Nuclear factor erythroid-2-related factor 2
**TWF1**	Twinfilin 1
**HCP5**	HLA complex P5
**SNHG8**	Small nucleolar RNA host gene 8
**TRAIL**	Tumor necrosis factor-related apoptosis inducing ligand
**DR5**	Death receptor 5
**EZH2**	Epigenetic regulator enhancer of zeste homolog 2
**CTBP1**	C-terminal binding protein 1
**NEAT1**	Nuclear paraspeckle assembly transcript 1
**PAK1**	P21 activated kinase 1
**MSC**	Mesenchymal stem cell
**SNHG7**	Small nucleolar RNA host gene 7
**SOCS3**	Suppressor of cytokine signaling 3
**GAS5**	Growth arrest-specific 5
**DLEU2L**	Deleted in lymphocytic leukemia 2-like
**SIRT1**	Sirtuin1
**LIF**	Leukemia inhibitory factor
**ESRP1**	Epithelial splicing regulatory protein 1
**siRNA**	Small interfering RNA
**shRNA**	Short hairpin RNA
**ASOs**	Anti-oligonucleotides
**CRISPR/Cas9**	Clustered regulatory interspaced short palindromic repeats-associated endonuclease 9

## References

[B1] Amponsah P. S. Fan P. Bauer N. Zhao Z. Gladkich J. Fellenberg J. (2017). microRNA-210 overexpression inhibits tumor growth and potentially reverses gemcitabine resistance in pancreatic cancer. Cancer Lett. 388, 107–117. 10.1016/j.canlet.2016.11.035 27940128

[B2] An N. Cheng D. (2020). The long noncoding RNA HOST2 promotes gemcitabine resistance in human pancreatic cancer cells. Pathol. Oncol. Res. 26, 425–431. 10.1007/s12253-018-0486-5 30406400

[B3] An N. Zheng B. (2020). MiR-203a-3p inhibits pancreatic cancer cell proliferation, EMT, and apoptosis by regulating SLUG. Technol. Cancer Res. Treat. 19, 1533033819898729. 10.1177/1533033819898729 32301392 PMC7168776

[B4] Angelousi A. Kamp K. Kaltsatou M. O'toole D. Kaltsas G. De Herder W. (2017). Sequential everolimus and sunitinib treatment in pancreatic metastatic well-differentiated neuroendocrine tumours resistant to prior treatments. Neuroendocrinology 105, 394–402. 10.1159/000456035 28122378

[B5] Bhatti I. Lee A. James V. Hall R. I. Lund J. N. Tufarelli C. (2011). Knockdown of microRNA-21 inhibits proliferation and increases cell death by targeting programmed cell death 4 (PDCD4) in pancreatic ductal adenocarcinoma. J. Gastrointest. Surg. 15, 199–208. 10.1007/s11605-010-1381-x 21088996

[B6] Bhutia Y. D. Hung S. W. Krentz M. Patel D. Lovin D. Manoharan R. (2013). Differential processing of let-7a precursors influences RRM2 expression and chemosensitivity in pancreatic cancer: Role of LIN-28 and SET oncoprotein. PLoS One 8, e53436. 10.1371/journal.pone.0053436 23335963 PMC3546076

[B7] Binenbaum Y. Fridman E. Yaari Z. Milman N. Schroeder A. Ben David G. (2018). Transfer of miRNA in macrophage-derived exosomes induces drug resistance in pancreatic adenocarcinoma. Cancer Res. 78, 5287–5299. 10.1158/0008-5472.CAN-18-0124 30042153

[B8] Bray F. Ferlay J. Soerjomataram I. Siegel R. L. Torre L. A. Jemal A. (2018). Global cancer statistics 2018: GLOBOCAN estimates of incidence and mortality worldwide for 36 cancers in 185 countries. CA Cancer J. Clin. 68, 394–424. 10.3322/caac.21492 30207593

[B9] Cai B. An Y. Lv N. Chen J. Tu M. Sun J. (2013). miRNA-181b increases the sensitivity of pancreatic ductal adenocarcinoma cells to gemcitabine *in vitro* and in nude mice by targeting BCL-2. Oncol. Rep. 29, 1769–1776. 10.3892/or.2013.2297 23440261

[B10] Carotenuto P. Amato F. Lampis A. Rae C. Hedayat S. Previdi M. C. (2021). Modulation of pancreatic cancer cell sensitivity to FOLFIRINOX through microRNA-mediated regulation of DNA damage. Nat. Commun. 12, 6738. 10.1038/s41467-021-27099-6 34795259 PMC8602334

[B11] Chaudhary A. K. Mondal G. Kumar V. Kattel K. Mahato R. I. (2017). Chemosensitization and inhibition of pancreatic cancer stem cell proliferation by overexpression of microRNA-205. Cancer Lett. 402, 1–8. 10.1016/j.canlet.2017.05.007 28536008 PMC5673079

[B12] Chen M. Wang M. Xu S. Guo X. Jiang J. (2015). Upregulation of miR-181c contributes to chemoresistance in pancreatic cancer by inactivating the Hippo signaling pathway. Oncotarget 6, 44466–44479. 10.18632/oncotarget.6298 26561204 PMC4792569

[B13] Chen W. Zhou Y. Zhi X. Ma T. Liu H. Chen B. W. (2019). Delivery of miR-212 by chimeric peptide-condensed supramolecular nanoparticles enhances the sensitivity of pancreatic ductal adenocarcinoma to doxorubicin. Biomaterials 192, 590–600. 10.1016/j.biomaterials.2018.11.035 30553134

[B14] Chen Y. Li Z. Zhang M. Wang B. Ye J. Zhang Y. (2019). Circ-ASH2L promotes tumor progression by sponging miR-34a to regulate Notch1 in pancreatic ductal adenocarcinoma. J. Exp. Clin. Cancer Res. 38, 466. 10.1186/s13046-019-1436-0 31718694 PMC6852927

[B15] Chen Z. W. Hu J. F. Wang Z. W. Liao C. Y. Kang F. P. Lin C. F. (2022). Circular RNA circ-MTHFD1L induces HR repair to promote gemcitabine resistance via the miR-615-3p/RPN6 axis in pancreatic ductal adenocarcinoma. J. Exp. Clin. Cancer Res. 41, 153. 10.1186/s13046-022-02343-z 35459186 PMC9034615

[B16] Cheng D. Fan J. Qin K. Zhou Y. Yang J. Ma Y. (2021). LncRNA SNHG7 regulates mesenchymal stem cell through the notch1/jagged1/hes-1 signaling pathway and influences folfirinox resistance in pancreatic cancer. Front. Oncol. 11, 719855. 10.3389/fonc.2021.719855 34631547 PMC8494469

[B17] Chi Y. Xin H. Liu Z. (2021). Exosomal lncRNA UCA1 derived from pancreatic stellate cells promotes gemcitabine resistance in pancreatic cancer via the SOCS3/EZH2 Axis. Front. Oncol. 11, 671082. 10.3389/fonc.2021.671082 34868904 PMC8640181

[B18] Cioffi M. Trabulo S. M. Sanchez-Ripoll Y. Miranda-Lorenzo I. Lonardo E. Dorado J. (2015). The miR-17-92 cluster counteracts quiescence and chemoresistance in a distinct subpopulation of pancreatic cancer stem cells. Gut 64, 1936–1948. 10.1136/gutjnl-2014-308470 25887381 PMC4680182

[B19] Deng Z. Li X. Shi Y. Lu Y. Yao W. Wang J. (2020). A novel autophagy-related IncRNAs signature for prognostic prediction and clinical value in patients with pancreatic cancer. Front. Cell Dev. Biol. 8, 606817. 10.3389/fcell.2020.606817 33384999 PMC7769875

[B20] Dhayat S. A. Mardin W. A. Seggewiss J. Strose A. J. Matuszcak C. Hummel R. (2015). MicroRNA profiling implies new markers of gemcitabine chemoresistance in mutant p53 pancreatic ductal adenocarcinoma. PLoS One 10, e0143755. 10.1371/journal.pone.0143755 26606261 PMC4659591

[B21] Dong J. Zhao Y. P. Zhou L. Zhang T. P. Chen G. (2011). Bcl-2 upregulation induced by miR-21 via a direct interaction is associated with apoptosis and chemoresistance in MIA PaCa-2 pancreatic cancer cells. Arch. Med. Res. 42, 8–14. 10.1016/j.arcmed.2011.01.006 21376256

[B22] Du J. He Y. Wu W. Li P. Chen Y. Hu Z. (2019). Targeting EphA2 with miR-124 mediates Erlotinib resistance in K-RAS mutated pancreatic cancer. J. Pharm. Pharmacol. 71, 196–205. 10.1111/jphp.12941 30604411

[B23] Fan P. Liu L. Yin Y. Zhao Z. Zhang Y. Amponsah P. S. (2016). MicroRNA-101-3p reverses gemcitabine resistance by inhibition of ribonucleotide reductase M1 in pancreatic cancer. Cancer Lett. 373, 130–137. 10.1016/j.canlet.2016.01.038 26828016

[B24] Fang Y. Zhou W. Rong Y. Kuang T. Xu X. Wu W. (2019). Exosomal miRNA-106b from cancer-associated fibroblast promotes gemcitabine resistance in pancreatic cancer. Exp. Cell Res. 383, 111543. 10.1016/j.yexcr.2019.111543 31374207

[B25] Feng Y. Gao L. Cui G. Cao Y. (2020). LncRNA NEAT1 facilitates pancreatic cancer growth and metastasis through stabilizing ELF3 mRNA. Am. J. Cancer Res. 10, 237–248. 32064164 PMC7017733

[B26] Franses J. W. Philipp J. Missios P. Bhan I. Liu A. Yashaswini C. (2020). Pancreatic circulating tumor cell profiling identifies LIN28B as a metastasis driver and drug target. Nat. Commun. 11, 3303. 10.1038/s41467-020-17150-3 32620742 PMC7335061

[B27] Funamizu N. Lacy C. R. Kamada M. Yanaga K. Manome Y. (2019). MicroRNA-200b and -301 are associated with gemcitabine response as biomarkers in pancreatic carcinoma cells. Int. J. Oncol. 54, 991–1000. 10.3892/ijo.2019.4676 30628651

[B28] Gao Y. Zhang Z. Li K. Gong L. Yang Q. Huang X. (2017). Linc-DYNC2H1-4 promotes EMT and CSC phenotypes by acting as a sponge of miR-145 in pancreatic cancer cells. Cell Death Dis. 8, e2924. 10.1038/cddis.2017.311 28703793 PMC5550858

[B29] Gao Z. Q. Wang J. F. Chen D. H. Ma X. S. Yang W. Zhe T. (2018). Long non-coding RNA GAS5 antagonizes the chemoresistance of pancreatic cancer cells through down-regulation of miR-181c-5p. Biomed. Pharmacother. 97, 809–817. 10.1016/j.biopha.2017.10.157 29112934

[B30] Giovannetti E. Funel N. Peters G. J. Del Chiaro M. Erozenci L. A. Vasile E. (2010). MicroRNA-21 in pancreatic cancer: Correlation with clinical outcome and pharmacologic aspects underlying its role in the modulation of gemcitabine activity. Cancer Res. 70, 4528–4538. 10.1158/0008-5472.CAN-09-4467 20460539

[B31] Gong Y. Dai H. S. Shu J. J. Liu W. Bie P. Zhang L. D. (2020). LNC00673 suppresses proliferation and metastasis of pancreatic cancer via target miR-504/HNF1A. J. Cancer 11, 940–948. 10.7150/jca.32855 31949497 PMC6959011

[B32] Gu J. Huang W. Wang X. Zhang J. Tao T. Zheng Y. (2022). Hsa-miR-3178/RhoB/PI3K/Akt, a novel signaling pathway regulates ABC transporters to reverse gemcitabine resistance in pancreatic cancer. Mol. Cancer 21, 112. 10.1186/s12943-022-01587-9 35538494 PMC9088115

[B33] Guo S. Fesler A. Huang W. Wang Y. Yang J. Wang X. (2020). Functional significance and therapeutic potential of miR-15a mimic in pancreatic ductal adenocarcinoma. Mol. Ther. Nucleic Acids 19, 228–239. 10.1016/j.omtn.2019.11.010 31846800 PMC6921186

[B34] Halfdanarson T. R. Foster N. R. Kim G. P. Meyers J. P. Smyrk T. C. Mccullough A. E. (2019). A phase II randomized trial of panitumumab, erlotinib, and gemcitabine versus erlotinib and gemcitabine in patients with untreated, metastatic pancreatic adenocarcinoma: North central cancer treatment group trial N064B (alliance). Oncologist 24, 589–e160. 10.1634/theoncologist.2018-0878 30679315 PMC6516109

[B35] Hamada S. Masamune A. Miura S. Satoh K. Shimosegawa T. (2014). MiR-365 induces gemcitabine resistance in pancreatic cancer cells by targeting the adaptor protein SHC1 and pro-apoptotic regulator BAX. Cell Signal 26, 179–185. 10.1016/j.cellsig.2013.11.003 24216611

[B36] Hao L. Rong W. Bai L. Cui H. Zhang S. Li Y. (2019). Upregulated circular RNA circ_0007534 indicates an unfavorable prognosis in pancreatic ductal adenocarcinoma and regulates cell proliferation, apoptosis, and invasion by sponging miR-625 and miR-892b. J. Cell Biochem. 120, 3780–3789. 10.1002/jcb.27658 30382592

[B37] Hasegawa S. Eguchi H. Nagano H. Konno M. Tomimaru Y. Wada H. (2014). MicroRNA-1246 expression associated with CCNG2-mediated chemoresistance and stemness in pancreatic cancer. Br. J. Cancer 111, 1572–1580. 10.1038/bjc.2014.454 25117811 PMC4200094

[B38] He J. Li F. Zhou Y. Hou X. Liu S. Li X. (2020). LncRNA XLOC_006390 promotes pancreatic carcinogenesis and glutamate metabolism by stabilizing c-Myc. Cancer Lett. 469, 419–428. 10.1016/j.canlet.2019.11.021 31734356

[B39] Hiramoto H. Muramatsu T. Ichikawa D. Tanimoto K. Yasukawa S. Otsuji E. (2017). miR-509-5p and miR-1243 increase the sensitivity to gemcitabine by inhibiting epithelial-mesenchymal transition in pancreatic cancer. Sci. Rep. 7, 4002. 10.1038/s41598-017-04191-w 28638102 PMC5479822

[B40] Hong C. Lishan W. Peng X. Zhengqing L. Yang Y. Fangfang H. (2022). Hsa_circ_0074298 promotes pancreatic cancer progression and resistance to gemcitabine by sponging miR-519 to target SMOC. J. Cancer 13, 34–50. 10.7150/jca.62927 34976169 PMC8692684

[B41] Houthuijzen J. M. Daenen L. G. Roodhart J. M. Voest E. E. (2012). The role of mesenchymal stem cells in anti-cancer drug resistance and tumour progression. Br. J. Cancer 106, 1901–1906. 10.1038/bjc.2012.201 22596239 PMC3388567

[B42] Hu C. Xia R. Zhang X. Li T. Ye Y. Li G. (2022). circFARP1 enables cancer-associated fibroblasts to promote gemcitabine resistance in pancreatic cancer via the LIF/STAT3 axis. Mol. Cancer 21, 24. 10.1186/s12943-022-01501-3 35045883 PMC8767726

[B43] Hu H. He Y. Wang Y. Chen W. Hu B. Gu Y. (2017). micorRNA-101 silences DNA-PKcs and sensitizes pancreatic cancer cells to gemcitabine. Biochem. Biophys. Res. Commun. 483, 725–731. 10.1016/j.bbrc.2016.12.074 27988337

[B44] Hu W. Liu Q. Pan J. Sui Z. (2018). MiR-373-3p enhances the chemosensitivity of gemcitabine through cell cycle pathway by targeting CCND2 in pancreatic carcinoma cells. Biomed. Pharmacother. 105, 887–898. 10.1016/j.biopha.2018.05.091 30021382

[B45] HuaY. Q.ZhuY. D.XieG. Q.ZhangK.ShengJ.ZhuZ. F. (2019). Long non-coding SBF2-AS1 acting as a competing endogenous RNA to sponge microRNA-142-3p to participate in gemcitabine resistance in pancreatic cancer via upregulating TWF1. Aging (Albany NY) 11, 8860–8878. 10.18632/aging.102307 31619579

[B46] Huang H. Li X. Zhang X. Li Z. Han D. Gao W. (2022). DSCR9/miR-21-5p axis inhibits pancreatic cancer proliferation and resistance to gemcitabine via BTG2 signaling. Acta Biochim. Biophys. Sin. (Shanghai) 54, 1775–1788. 10.3724/abbs.2022194 36789695 PMC10157615

[B47] Huang H. Xiong G. Shen P. Cao Z. Zheng L. Zhang T. (2017). MicroRNA-1285 inhibits malignant biological behaviors of human pancreatic cancer cells by negative regulation of YAP1. Neoplasma 64, 358–366. 10.4149/neo_2017_306 28253715

[B48] Huang L. Hu C. Cao H. Wu X. Wang R. Lu H. (2018). MicroRNA-29c increases the chemosensitivity of pancreatic cancer cells by inhibiting USP22 mediated autophagy. Cell Physiol. Biochem. 47, 747–758. 10.1159/000490027 29807360

[B49] Huang L. J. Shen Y. Bai J. Wang F. X. Feng Y. D. Chen H. L. (2020). High expression levels of long noncoding RNA small nucleolar RNA host gene 18 and semaphorin 5A indicate poor prognosis in multiple myeloma. Acta Haematol. 143, 279–288. 10.1159/000502404 31597158

[B50] Huang R. Song X. Wang C. M. (2019). MiR-223 regulates CDDP resistance in pancreatic cancer via targeting FoxO3a. Eur. Rev. Med. Pharmacol. Sci. 23, 7892–7898. 10.26355/eurrev_201909_19000 31599414

[B51] Hwang J. H. Voortman J. Giovannetti E. Steinberg S. M. Leon L. G. Kim Y. T. (2010). Identification of microRNA-21 as a biomarker for chemoresistance and clinical outcome following adjuvant therapy in resectable pancreatic cancer. PLoS One 5, e10630. 10.1371/journal.pone.0010630 20498843 PMC2871055

[B52] Irigoyen A. Gallego J. Guillen Ponce C. Vera R. Iranzo V. Ales I. (2017). Gemcitabine-erlotinib versus gemcitabine-erlotinib-capecitabine in the first-line treatment of patients with metastatic pancreatic cancer: Efficacy and safety results of a phase IIb randomised study from the Spanish TTD Collaborative Group. Eur. J. Cancer 75, 73–82. 10.1016/j.ejca.2016.12.032 28222309

[B53] Iwagami Y. Eguchi H. Nagano H. Akita H. Hama N. Wada H. (2013). miR-320c regulates gemcitabine-resistance in pancreatic cancer via SMARCC1. Br. J. Cancer 109, 502–511. 10.1038/bjc.2013.320 23799850 PMC3721395

[B54] Izumchenko E. Chang X. Michailidi C. Kagohara L. Ravi R. Paz K. (2014). The TGFβ-miR200-MIG6 pathway orchestrates the EMT-associated kinase switch that induces resistance to EGFR inhibitors. Cancer Res. 74, 3995–4005. 10.1158/0008-5472.CAN-14-0110 24830724 PMC4122100

[B55] Jadeja R. N. Jones M. A. Abdelrahman A. A. Powell F. L. Thounaojam M. C. Gutsaeva D. (2020). Inhibiting microRNA-144 potentiates Nrf2-dependent antioxidant signaling in RPE and protects against oxidative stress-induced outer retinal degeneration. Redox Biol. 28, 101336. 10.1016/j.redox.2019.101336 31590045 PMC6812120

[B56] Ji Q. Hao X. Zhang M. Tang W. Yang M. Li L. (2009). MicroRNA miR-34 inhibits human pancreatic cancer tumor-initiating cells. PLoS One 4, e6816. 10.1371/journal.pone.0006816 19714243 PMC2729376

[B57] Jia L. Xi Q. Wang H. Zhang Z. Liu H. Cheng Y. (2017). miR-142-5p regulates tumor cell PD-L1 expression and enhances anti-tumor immunity. Biochem. Biophys. Res. Commun. 488, 425–431. 10.1016/j.bbrc.2017.05.074 28511795

[B58] Jiang M. J. Chen Y. Y. Dai J. J. Gu D. N. Mei Z. Liu F. R. (2020). Dying tumor cell-derived exosomal miR-194-5p potentiates survival and repopulation of tumor repopulating cells upon radiotherapy in pancreatic cancer. Mol. Cancer 19, 68. 10.1186/s12943-020-01178-6 32228703 PMC7104536

[B59] Jiang W. Zhao S. Shen J. Guo L. Sun Y. Zhu Y. (2018). The MiR-135b-BMAL1-YY1 loop disturbs pancreatic clockwork to promote tumourigenesis and chemoresistance. Cell Death Dis. 9, 149. 10.1038/s41419-017-0233-y 29396463 PMC5833454

[B60] Jiao F. Hu H. Han T. Yuan C. Wang L. Jin Z. (2015). Long noncoding RNA MALAT-1 enhances stem cell-like phenotypes in pancreatic cancer cells. Int. J. Mol. Sci. 16, 6677–6693. 10.3390/ijms16046677 25811929 PMC4424983

[B61] Johnsson J. (1991). Developing a winning strategy for managed care contracting. Hospitals 65, 26–28. 1894271

[B62] Kamath S. D. Kalyan A. Kircher S. Nimeiri H. Fought A. J. Benson A. 3rd (2020). Ipilimumab and gemcitabine for advanced pancreatic cancer: A phase ib study. Oncologist 25, e808–e815. 10.1634/theoncologist.2019-0473 31740568 PMC7216436

[B63] Kim K. H. Lee M. S. (2014). Autophagy-a key player in cellular and body metabolism. Nat. Rev. Endocrinol. 10, 322–337. 10.1038/nrendo.2014.35 24663220

[B64] Knudsen E. S. Kumarasamy V. Ruiz A. Sivinski J. Chung S. Grant A. (2019). Cell cycle plasticity driven by MTOR signaling: Integral resistance to CDK4/6 inhibition in patient-derived models of pancreatic cancer. Oncogene 38, 3355–3370. 10.1038/s41388-018-0650-0 30696953 PMC6499706

[B65] Kristensen L. S. Andersen M. S. Stagsted L. V. W. Ebbesen K. K. Hansen T. B. Kjems J. (2019). The biogenesis, biology and characterization of circular RNAs. Nat. Rev. Genet. 20, 675–691. 10.1038/s41576-019-0158-7 31395983

[B66] Kurtanich T. Roos N. Wang G. Yang J. Wang A. Chung E. J. (2019). Pancreatic cancer gene therapy delivered by nanoparticles. SLAS Technol. 24, 151–160. 10.1177/2472630318811108 30395768

[B67] Kuwada K. Kagawa S. Yoshida R. Sakamoto S. Ito A. Watanabe M. (2018). The epithelial-to-mesenchymal transition induced by tumor-associated macrophages confers chemoresistance in peritoneally disseminated pancreatic cancer. J. Exp. Clin. Cancer Res. 37, 307. 10.1186/s13046-018-0981-2 30537992 PMC6288926

[B68] Lee J. T. (2012). Epigenetic regulation by long noncoding RNAs. Science 338, 1435–1439. 10.1126/science.1231776 23239728

[B69] Levy J. M. M. Towers C. G. Thorburn A. (2017). Targeting autophagy in cancer. Nat. Rev. Cancer 17, 528–542. 10.1038/nrc.2017.53 28751651 PMC5975367

[B70] Li J. Wu H. Li W. Yin L. Guo S. Xu X. (2016). Downregulated miR-506 expression facilitates pancreatic cancer progression and chemoresistance via SPHK1/Akt/NF-κB signaling. Oncogene 35, 5501–5514. 10.1038/onc.2016.90 27065335 PMC5078861

[B71] Li M. Li H. Chen Q. Wu W. Chen X. Ran L. (2020a). A novel and robust long noncoding RNA panel to predict the prognosis of pancreatic cancer. DNA Cell Biol. 39, 1282–1289. 10.1089/dna.2019.5241 32522048

[B72] Li M. Wu P. Yang Z. Deng S. Ni L. Zhang Y. (2020b). miR-193a-5p promotes pancreatic cancer cell metastasis through SRSF6-mediated alternative splicing of OGDHL and ECM1. Am. J. Cancer Res. 10, 38–59. 32064152 PMC7017744

[B73] Li Z. Li X. Yu C. Wang M. Peng F. Xiao J. (2014). MicroRNA-100 regulates pancreatic cancer cells growth and sensitivity to chemotherapy through targeting FGFR3. Tumour Biol. 35, 11751–11759. 10.1007/s13277-014-2271-8 25344675

[B74] Li Z. Zhao X. Zhou Y. Liu Y. Zhou Q. Ye H. (2015). The long non-coding RNA HOTTIP promotes progression and gemcitabine resistance by regulating HOXA13 in pancreatic cancer. J. Transl. Med. 13, 84. 10.1186/s12967-015-0442-z 25889214 PMC4372045

[B75] Liang C. Wang Z. Li Y. Y. Yu B. H. Zhang F. Li H. Y. (2015a). miR-33a suppresses the nuclear translocation of beta-catenin to enhance gemcitabine sensitivity in human pancreatic cancer cells. Tumour Biol. 36, 9395–9403. 10.1007/s13277-015-3679-5 26113407

[B76] Liang C. Yu X. J. Guo X. Z. Sun M. H. Wang Z. Song Y. (2015b). MicroRNA-33a-mediated downregulation of Pim-3 kinase expression renders human pancreatic cancer cells sensitivity to gemcitabine. Oncotarget 6, 14440–14455. 10.18632/oncotarget.3885 25971209 PMC4546478

[B77] Lin C. Wang Y. Dong Y. Lai S. Wang L. Weng S. (2022). N6-methyladenosine-mediated SH3BP5-AS1 upregulation promotes GEM chemoresistance in pancreatic cancer by activating the Wnt signaling pathway. Biol. Direct 17, 33. 10.1186/s13062-022-00347-5 36397058 PMC9673340

[B78] Lin Y. Ge X. Wen Y. Shi Z. M. Chen Q. D. Wang M. (2016). MiRNA-145 increases therapeutic sensibility to gemcitabine treatment of pancreatic adenocarcinoma cells. Oncotarget 7, 70857–70868. 10.18632/oncotarget.12268 27765914 PMC5342594

[B79] Lin Z. Lu S. Xie X. Yi X. Huang H. (2020). Noncoding RNAs in drug-resistant pancreatic cancer: A review. Biomed. Pharmacother. 131, 110768. 10.1016/j.biopha.2020.110768 33152930

[B80] Liu A. Zhou Y. Zhao T. Tang X. Zhou B. Xu J. (2021). MiRNA-3662 reverses the gemcitabine resistance in pancreatic cancer through regulating the tumor metabolism. Cancer Chemother. Pharmacol. 88, 343–357. 10.1007/s00280-021-04289-z 33993382

[B81] Liu B. Wu S. Ma J. Yan S. Xiao Z. Wan L. (2018). lncRNA GAS5 reverses EMT and tumor stem cell-mediated gemcitabine resistance and metastasis by targeting miR-221/SOCS3 in pancreatic cancer. Mol. Ther. Nucleic Acids 13, 472–482. 10.1016/j.omtn.2018.09.026 30388621 PMC6205337

[B82] Liu C. Billet S. Choudhury D. Cheng R. Haldar S. Fernandez A. (2021). Bone marrow mesenchymal stem cells interact with head and neck squamous cell carcinoma cells to promote cancer progression and drug resistance. Neoplasia 23, 118–128. 10.1016/j.neo.2020.11.012 33310208 PMC7732973

[B83] Liu F. Liu B. Qian J. Wu G. Li J. Ma Z. (2017). miR-153 enhances the therapeutic effect of gemcitabine by targeting Snail in pancreatic cancer. Acta Biochim. Biophys. Sin. (Shanghai) 49, 520–529. 10.1093/abbs/gmx039 28459992

[B84] Liu G. Ji L. Ke M. Ou Z. Tang N. Li Y. (2018). miR-125a-3p is responsible for chemosensitivity in PDAC by inhibiting epithelial-mesenchymal transition via Fyn. Biomed. Pharmacother. 106, 523–531. 10.1016/j.biopha.2018.06.114 29990840

[B85] Liu Q. G. Li Y. J. Yao L. (2018). Knockdown of AGR2 induces cell apoptosis and reduces chemotherapy resistance of pancreatic cancer cells with the involvement of ERK/AKT axis. Pancreatology 18, 678–688. 10.1016/j.pan.2018.07.003 30055941

[B86] Liu S. L. Cai C. Yang Z. Y. Wu Z. Y. Wu X. S. Wang X. F. (2021). DGCR5 is activated by PAX5 and promotes pancreatic cancer via targeting miR-3163/TOP2A and activating Wnt/β-catenin pathway. Int. J. Biol. Sci. 17, 498–513. 10.7150/ijbs.55636 33613108 PMC7893588

[B87] Liu Y. F. Luo D. Li X. Li Z. Q. Yu X. Zhu H. W. (2021). PVT1 knockdown inhibits autophagy and improves gemcitabine sensitivity by regulating the MiR-143/HIF-1α/VMP1 Axis in pancreatic cancer. Pancreas 50, 227–234. 10.1097/MPA.0000000000001747 33565800

[B88] Liu Y. Li X. Zhu S. Zhang J. G. Yang M. Qin Q. (2015). Ectopic expression of miR-494 inhibited the proliferation, invasion and chemoresistance of pancreatic cancer by regulating SIRT1 and c-Myc. Gene Ther. 22, 729–738. 10.1038/gt.2015.39 25965392

[B89] Liu Y. Wang J. Dong L. Xia L. Zhu H. Li Z. (2019). Long noncoding RNA HCP5 regulates pancreatic cancer gemcitabine (GEM) resistance by sponging hsa-miR-214-3p to target HDGF. Onco Targets Ther. 12, 8207–8216. 10.2147/OTT.S222703 31632071 PMC6781945

[B90] Liu Y. Xia L. Dong L. Wang J. Xiao Q. Yu X. (2020). CircHIPK3 promotes gemcitabine (GEM) resistance in pancreatic cancer cells by sponging miR-330-5p and targets RASSF1. Cancer Manag. Res. 12, 921–929. 10.2147/CMAR.S239326 32104074 PMC7023912

[B91] Lu H. Lu S. Yang D. Zhang L. Ye J. Li M. (2019). MiR-20a-5p regulates gemcitabine chemosensitivity by targeting RRM2 in pancreatic cancer cells and serves as a predictor for gemcitabine-based chemotherapy. Biosci. Rep. 39. 10.1042/BSR20181374 PMC6504660 30777929

[B92] Lu Y. Zhou S. Cheng G. Ruan Y. Tian Y. Lv K. (2022). CircLMTK2 silencing attenuates gemcitabine resistance in pancreatic cancer by sponging miR-485-5p and to target PAK1. J. Oncol. 2022, 1911592. 10.1155/2022/1911592 36059806 PMC9433304

[B93] Luo G. Xia X. Wang X. Zhang K. Cao J. Jiang T. (2018). miR-301a plays a pivotal role in hypoxia-induced gemcitabine resistance in pancreatic cancer. Exp. Cell Res. 369, 120–128. 10.1016/j.yexcr.2018.05.013 29772221

[B94] Luo M. Deng X. Chen Z. Hu Y. (2023). Circular RNA circPOFUT1 enhances malignant phenotypes and autophagy-associated chemoresistance via sequestrating miR-488-3p to activate the PLAG1-ATG12 axis in gastric cancer. Cell Death Dis. 14, 10. 10.1038/s41419-022-05506-0 36624091 PMC9829716

[B95] Ma C. Huang T. Ding Y. C. Yu W. Wang Q. Meng B. (2015). MicroRNA-200c overexpression inhibits chemoresistance, invasion and colony formation of human pancreatic cancer stem cells. Int. J. Clin. Exp. Pathol. 8, 6533–6539. 26261532 PMC4525866

[B96] Ma L. Fan Z. Du G. Wang H. (2019). Leptin-elicited miRNA-342-3p potentiates gemcitabine resistance in pancreatic ductal adenocarcinoma. Biochem. Biophys. Res. Commun. 509, 845–853. 10.1016/j.bbrc.2019.01.030 30638935

[B97] Ma L. Wang F. Du C. Zhang Z. Guo H. Xie X. (2018). Long non-coding RNA MEG3 functions as a tumour suppressor and has prognostic predictive value in human pancreatic cancer. Oncol. Rep. 39, 1132–1140. 10.3892/or.2018.6178 29328401

[B98] Ma T. Chen W. Zhi X. Liu H. Zhou Y. Chen B. W. (2018). USP9X inhibition improves gemcitabine sensitivity in pancreatic cancer by inhibiting autophagy. Cancer Lett. 436, 129–138. 10.1016/j.canlet.2018.08.010 30118840

[B99] Maftouh M. Avan A. Funel N. Frampton A. E. Fiuji H. Pelliccioni S. (2014). miR-211 modulates gemcitabine activity through downregulation of ribonucleotide reductase and inhibits the invasive behavior of pancreatic cancer cells. Nucleosides Nucleotides Nucleic Acids 33, 384–393. 10.1080/15257770.2014.891741 24940696

[B100] Makler A. Asghar W. (2023). Exosomal miRNA biomarker panel for pancreatic ductal adenocarcinoma detection in patient plasma: A pilot study. Int. J. Mol. Sci. 24, 5081. 10.3390/ijms24065081 36982154 PMC10049393

[B101] Meijer L. L. Garajova I. Caparello C. Le Large T. Y. S. Frampton A. E. Vasile E. (2020). Plasma miR-181a-5p downregulation predicts response and improved survival after FOLFIRINOX in pancreatic ductal adenocarcinoma. Ann. Surg. 271, 1137–1147. 10.1097/SLA.0000000000003084 30394883

[B102] Meng Q. Liang C. Hua J. Zhang B. Liu J. Zhang Y. (2020). A miR-146a-5p/TRAF6/NF-kB p65 axis regulates pancreatic cancer chemoresistance: Functional validation and clinical significance. Theranostics 10, 3967–3979. 10.7150/thno.40566 32226532 PMC7086345

[B103] Mikamori M. Yamada D. Eguchi H. Hasegawa S. Kishimoto T. Tomimaru Y. (2017). MicroRNA-155 controls exosome synthesis and promotes gemcitabine resistance in pancreatic ductal adenocarcinoma. Sci. Rep. 7, 42339. 10.1038/srep42339 28198398 PMC5309735

[B104] Miyamae M. Komatsu S. Ichikawa D. Kawaguchi T. Hirajima S. Okajima W. (2015). Plasma microRNA profiles: Identification of miR-744 as a novel diagnostic and prognostic biomarker in pancreatic cancer. Br. J. Cancer 113, 1467–1476. 10.1038/bjc.2015.366 26505678 PMC4815891

[B105] Modi S. Kir D. Giri B. Majumder K. Arora N. Dudeja V. (2016). Minnelide overcomes oxaliplatin resistance by downregulating the DNA repair pathway in pancreatic cancer. J. Gastrointest. Surg. 20, 13–23. 10.1007/s11605-015-3000-3 26503259 PMC4698020

[B106] Nagano H. Tomimaru Y. Eguchi H. Hama N. Wada H. Kawamoto K. (2013). MicroRNA-29a induces resistance to gemcitabine through the Wnt/β-catenin signaling pathway in pancreatic cancer cells. Int. J. Oncol. 43, 1066–1072. 10.3892/ijo.2013.2037 23900458

[B107] Nguyen L. Schilling D. Dobiasch S. Raulefs S. Santiago Franco M. Buschmann D. (2020). The emerging role of miRNAs for the radiation treatment of pancreatic cancer. Cancers (Basel) 12, 3703. 10.3390/cancers12123703 33317198 PMC7763922

[B108] Nunez J. E. Donadio M. Filho D. R. Rego J. F. Barros M. Formiga M. N. (2019). The efficacy of everolimus and sunitinib in patients with sporadic or germline mutated metastatic pancreatic neuroendocrine tumors. J. Gastrointest. Oncol. 10, 645–651. 10.21037/jgo.2019.01.33 31392045 PMC6657324

[B109] Ohuchida K. Mizumoto K. Kayashima T. Fujita H. Moriyama T. Ohtsuka T. (2011). MicroRNA expression as a predictive marker for gemcitabine response after surgical resection of pancreatic cancer. Ann. Surg. Oncol. 18, 2381–2387. 10.1245/s10434-011-1602-x 21347785 PMC3136688

[B110] Okazaki J. Tanahashi T. Sato Y. Miyoshi J. Nakagawa T. Kimura T. (2020). MicroRNA-296-5p promotes cell invasion and drug resistance by targeting bcl2-related ovarian killer, leading to a poor prognosis in pancreatic cancer. Digestion 101, 794–806. 10.1159/000503225 31563901

[B111] Ouyang L. Liu R. D. Lei D. Q. Shang Q. C. Li H. F. Hu X. G. (2021). MiR-499a-5p promotes 5-FU resistance and the cell proliferation and migration through activating PI3K/Akt signaling by targeting PTEN in pancreatic cancer. Ann. Transl. Med. 9, 1798. 10.21037/atm-21-6556 35071492 PMC8756217

[B112] Pammer J. Rossiter H. Bilban M. Eckhart L. Buchberger M. Monschein L. (2020). PIWIL-2 and piRNAs are regularly expressed in epithelia of the skin and their expression is related to differentiation. Arch. Dermatol Res. 312, 705–714. 10.1007/s00403-020-02052-7 32166374 PMC7548280

[B113] Pandya G. Kirtonia A. Sethi G. Pandey A. K. Garg M. (2020). The implication of long non-coding RNAs in the diagnosis, pathogenesis and drug resistance of pancreatic ductal adenocarcinoma and their possible therapeutic potential. Biochim. Biophys. Acta Rev. Cancer 1874, 188423. 10.1016/j.bbcan.2020.188423 32871244

[B114] Panebianco C. Trivieri N. Villani A. Terracciano F. Latiano T. P. Potenza A. (2021). Improving gemcitabine sensitivity in pancreatic cancer cells by restoring miRNA-217 levels. Biomolecules 11, 639. 10.3390/biom11050639 33925948 PMC8146031

[B115] Park J. K. Lee E. J. Esau C. Schmittgen T. D. (2009). Antisense inhibition of microRNA-21 or -221 arrests cell cycle, induces apoptosis, and sensitizes the effects of gemcitabine in pancreatic adenocarcinoma. Pancreas 38, e190–e199. 10.1097/MPA.0b013e3181ba82e1 19730150

[B116] Passadouro M. Pedroso De Lima M. C. Faneca H. (2014). MicroRNA modulation combined with sunitinib as a novel therapeutic strategy for pancreatic cancer. Int. J. Nanomedicine 9, 3203–3217. 10.2147/IJN.S64456 25061297 PMC4086670

[B117] Patel G. K. Khan M. A. Bhardwaj A. Srivastava S. K. Zubair H. Patton M. C. (2017). Exosomes confer chemoresistance to pancreatic cancer cells by promoting ROS detoxification and miR-155-mediated suppression of key gemcitabine-metabolising enzyme, DCK. Br. J. Cancer 116, 609–619. 10.1038/bjc.2017.18 28152544 PMC5344296

[B118] Previdi M. C. Carotenuto P. Zito D. Pandolfo R. Braconi C. (2017). Noncoding RNAs as novel biomarkers in pancreatic cancer: What do we know? Future Oncol. 13, 443–453. 10.2217/fon-2016-0253 27841659 PMC5253462

[B119] Qian Y. Xiong Y. Feng D. Wu Y. Zhang X. Chen L. (2019). Coix seed extract enhances the anti-pancreatic cancer efficacy of gemcitabine through regulating ABCB1- and ABCG2-mediated drug efflux: A bioluminescent pharmacokinetic and pharmacodynamic study. Int. J. Mol. Sci. 20, 5250. 10.3390/ijms20215250 31652737 PMC6862065

[B120] Qin G. Tu X. Li H. Cao P. Chen X. Song J. (2020). Long noncoding RNA p53-stabilizing and activating RNA promotes p53 signaling by inhibiting heterogeneous nuclear ribonucleoprotein K deSUMOylation and suppresses hepatocellular carcinoma. Hepatology 71, 112–129. 10.1002/hep.30793 31148184

[B121] Qin X. Guo H. Wang X. Zhu X. Yan M. Wang X. (2019). Exosomal miR-196a derived from cancer-associated fibroblasts confers cisplatin resistance in head and neck cancer through targeting CDKN1B and ING5. Genome Biol. 20, 12. 10.1186/s13059-018-1604-0 30642385 PMC6332863

[B122] Qu S. Niu K. Wang J. Dai J. Ganguly A. Gao C. (2021). LINC00671 suppresses cell proliferation and metastasis in pancreatic cancer by inhibiting AKT and ERK signaling pathway. Cancer Gene Ther. 28, 221–233. 10.1038/s41417-020-00213-4 32801328

[B123] Rajabpour A. Afgar A. Mahmoodzadeh H. Radfar J. E. Rajaei F. Teimoori-Toolabi L. (2017). MiR-608 regulating the expression of ribonucleotide reductase M1 and cytidine deaminase is repressed through induced gemcitabine chemoresistance in pancreatic cancer cells. Cancer Chemother. Pharmacol. 80, 765–775. 10.1007/s00280-017-3418-2 28887583

[B124] Schreiber R. Mezencev R. Matyunina L. V. Mcdonald J. F. (2016). Evidence for the role of microRNA 374b in acquired cisplatin resistance in pancreatic cancer cells. Cancer Gene Ther. 23, 241–245. 10.1038/cgt.2016.23 27229158 PMC5007605

[B125] Shao F. Huang M. Meng F. Huang Q. (2018). Circular RNA signature predicts gemcitabine resistance of pancreatic ductal adenocarcinoma. Front. Pharmacol. 9, 584. 10.3389/fphar.2018.00584 29922161 PMC5996282

[B126] Shen Y. Pan Y. Xu L. Chen L. Liu L. Chen H. (2015). Identifying microRNA-mRNA regulatory network in gemcitabine-resistant cells derived from human pancreatic cancer cells. Tumour Biol. 36, 4525–4534. 10.1007/s13277-015-3097-8 25722110

[B127] Shi W. Zhang C. Ning Z. Hua Y. Li Y. Chen L. (2019). Long non-coding RNA LINC00346 promotes pancreatic cancer growth and gemcitabine resistance by sponging miR-188-3p to derepress BRD4 expression. J. Exp. Clin. Cancer Res. 38, 60. 10.1186/s13046-019-1055-9 30728036 PMC6366022

[B128] Song B. Ye L. Wu S. Jing Z. (2020). Long non-coding RNA MEG3 regulates CSE-induced apoptosis and inflammation via regulating miR-218 in 16HBE cells. Biochem. Biophys. Res. Commun. 521, 368–374. 10.1016/j.bbrc.2019.10.135 31668807

[B129] Song Y. Zou L. Li J. Shen Z. P. Cai Y. L. Wu X. D. (2018). LncRNA SNHG8 promotes the development and chemo-resistance of pancreatic adenocarcinoma. Eur. Rev. Med. Pharmacol. Sci. 22, 8161–8168. 10.26355/eurrev_201812_16508 30556854

[B130] Springfeld C. Jager D. Buchler M. W. Strobel O. Hackert T. Palmer D. H. (2019). Chemotherapy for pancreatic cancer. Presse Med. 48, e159–e174. 10.1016/j.lpm.2019.02.025 30879894

[B131] Su Q. Liu Y. Lv X. W. Dai R. X. Yang X. H. Kong B. H. (2020). LncRNA TUG1 mediates ischemic myocardial injury by targeting miR-132-3p/HDAC3 axis. Am. J. Physiol. Heart Circ. Physiol. 318, H332–H344. 10.1152/ajpheart.00444.2019 31858814

[B132] Sun D. Wang X. Sui G. Chen S. Yu M. Zhang P. (2018). Downregulation of miR-374b-5p promotes chemotherapeutic resistance in pancreatic cancer by upregulating multiple anti-apoptotic proteins. Int. J. Oncol. 52, 1491–1503. 10.3892/ijo.2018.4315 29568910 PMC5873836

[B133] Takiuchi D. Eguchi H. Nagano H. Iwagami Y. Tomimaru Y. Wada H. (2013). Involvement of microRNA-181b in the gemcitabine resistance of pancreatic cancer cells. Pancreatology 13, 517–523. 10.1016/j.pan.2013.06.007 24075517

[B134] Tan P. Li M. Liu Z. Li T. Zhao L. Fu W. (2022). Glycolysis-related LINC02432/hsa-miR-98-5p/HK2 Axis inhibits ferroptosis and predicts immune infiltration, tumor mutation burden, and drug sensitivity in pancreatic adenocarcinoma. Front. Pharmacol. 13, 937413. 10.3389/fphar.2022.937413 35795552 PMC9251347

[B135] Tasaki Y. Suzuki M. Katsushima K. Shinjo K. Iijima K. Murofushi Y. (2021). Cancer-specific targeting of taurine-upregulated gene 1 enhances the effects of chemotherapy in pancreatic cancer. Cancer Res. 81, 1654–1666. 10.1158/0008-5472.CAN-20-3021 33648930

[B136] Tian X. Shivapurkar N. Wu Z. Hwang J. J. Pishvaian M. J. Weiner L. M. (2016). Circulating microRNA profile predicts disease progression in patients receiving second-line treatment of lapatinib and capecitabine for metastatic pancreatic cancer. Oncol. Lett. 11, 1645–1650. 10.3892/ol.2016.4101 26998056 PMC4774452

[B137] Tu M. J. Duan Z. Liu Z. Zhang C. Bold R. J. Gonzalez F. J. (2020). MicroRNA-1291-5p sensitizes pancreatic carcinoma cells to arginine deprivation and chemotherapy through the regulation of arginolysis and glycolysis. Mol. Pharmacol. 98, 686–694. 10.1124/molpharm.120.000130 33051382 PMC7673485

[B138] Von Hoff D. D. Korn R. Mousses S. (2009). Pancreatic cancer-could it be that simple? A different context of vulnerability. Cancer Cell 16, 7–8. 10.1016/j.ccr.2009.06.011 19573807

[B139] Wang L. Bi R. Li L. Zhou K. Yin H. (2021). lncRNA ANRIL aggravates the chemoresistance of pancreatic cancer cells to gemcitabine by targeting inhibition of miR-181a and targeting HMGB1-induced autophagy. Aging (Albany NY) 13, 19272–19281. 10.18632/aging.203251 34374662 PMC8386553

[B140] Wang L. Wang F. Na L. Yu J. Huang L. Meng Z. Q. (2018). LncRNA AB209630 inhibits gemcitabine resistance cell proliferation by regulating PI3K/AKT signaling in pancreatic ductal adenocarcinoma. Cancer Biomark. 22, 169–174. 10.3233/CBM-181182 29526843 PMC13078444

[B141] Wang P. Zhuang L. Zhang J. Fan J. Luo J. Chen H. (2013). The serum miR-21 level serves as a predictor for the chemosensitivity of advanced pancreatic cancer, and miR-21 expression confers chemoresistance by targeting FasL. Mol. Oncol. 7, 334–345. 10.1016/j.molonc.2012.10.011 23177026 PMC5528497

[B142] Wang T. Chen G. Ma X. Yang Y. Chen Y. Peng Y. (2019). MiR-30a regulates cancer cell response to chemotherapy through SNAI1/IRS1/AKT pathway. Cell Death Dis. 10, 153. 10.1038/s41419-019-1326-6 30770779 PMC6377638

[B143] Wang W. Zhao L. Wei X. Wang L. Liu S. Yang Y. (2016). MicroRNA-320a promotes 5-FU resistance in human pancreatic cancer cells. Sci. Rep. 6, 27641. 10.1038/srep27641 27279541 PMC4899709

[B144] Wang Y. Zhang Q. Guo B. Feng J. Zhao D. (2020). miR-1231 is downregulated in prostate cancer with prognostic and functional implications. Oncol. Res. Treat. 43, 78–86. 10.1159/000504606 31822000

[B145] Wang Z. C. Huang F. Z. Xu H. B. Sun J. C. Wang C. F. (2019). MicroRNA-137 inhibits autophagy and chemosensitizes pancreatic cancer cells by targeting ATG5. Int. J. Biochem. Cell Biol. 111, 63–71. 10.1016/j.biocel.2019.01.020 30710750

[B146] Wei X. Wang W. Wang L. Zhang Y. Zhang X. Chen M. (2016). MicroRNA-21 induces 5-fluorouracil resistance in human pancreatic cancer cells by regulating PTEN and PDCD4. Cancer Med. 5, 693–702. 10.1002/cam4.626 26864640 PMC4831288

[B147] Wei Y. Chen X. Liang C. Ling Y. Yang X. Ye X. (2020). A noncoding regulatory RNAs network driven by circ-CDYL acts specifically in the early stages hepatocellular carcinoma. Hepatology 71, 130–147. 10.1002/hep.30795 31148183

[B148] Weiss G. J. Blaydorn L. Beck J. Bornemann-Kolatzki K. Urnovitz H. Schutz E. (2018). Phase Ib/II study of gemcitabine, nab-paclitaxel, and pembrolizumab in metastatic pancreatic adenocarcinoma. Invest. New Drugs 36, 96–102. 10.1007/s10637-017-0525-1 29119276

[B149] Wong C. H. Lou U. K. Li Y. Chan S. L. Tong J. H. To K. F. (2020). CircFOXK2 promotes growth and metastasis of pancreatic ductal adenocarcinoma by complexing with RNA-binding proteins and sponging MiR-942. Cancer Res. 80, 2138–2149. 10.1158/0008-5472.CAN-19-3268 32217695

[B150] Wu M. Tan X. Liu P. Yang Y. Huang Y. Liu X. (2020). Role of exosomal microRNA-125b-5p in conferring the metastatic phenotype among pancreatic cancer cells with different potential of metastasis. Life Sci. 255, 117857. 10.1016/j.lfs.2020.117857 32470446

[B151] Wu M. Zhang P. (2020). EGFR-mediated autophagy in tumourigenesis and therapeutic resistance. Cancer Lett. 469, 207–216. 10.1016/j.canlet.2019.10.030 31639425

[B152] Wu R. Su Z. Zhao L. Pei R. Ding Y. Li D. (2023). Extracellular vesicle-loaded oncogenic lncRNA NEAT1 from adipose-derived mesenchymal stem cells confers gemcitabine resistance in pancreatic cancer via miR-491-5p/snail/SOCS3 Axis. Stem Cells Int. 2023, 6510571. 10.1155/2023/6510571 36762032 PMC9902843

[B153] Wu Y. Xu W. Yang Y. Zhang Z. (2021). miRNA-93-5p promotes gemcitabine resistance in pancreatic cancer cells by targeting the PTEN-mediated PI3K/Akt signaling pathway. Ann. Clin. Lab. Sci. 51, 310–320. 34162560

[B154] Xia X. Zhang K. Luo G. Cen G. Cao J. Huang K. (2017). Downregulation of miR-301a-3p sensitizes pancreatic cancer cells to gemcitabine treatment via PTEN. Am. J. Transl. Res. 9, 1886–1895. 28469793 PMC5411936

[B155] Xiao J. Peng F. Yu C. Wang M. Li X. Li Z. (2014). microRNA-137 modulates pancreatic cancer cells tumor growth, invasion and sensitivity to chemotherapy. Int. J. Clin. Exp. Pathol. 7, 7442–7450. 25550779 PMC4270551

[B156] Xiong G. Huang H. Feng M. Yang G. Zheng S. You L. (2018). MiR-10a-5p targets TFAP2C to promote gemcitabine resistance in pancreatic ductal adenocarcinoma. J. Exp. Clin. Cancer Res. 37, 76. 10.1186/s13046-018-0739-x 29615098 PMC5883523

[B157] Xiong G. Liu C. Yang G. Feng M. Xu J. Zhao F. (2019). Long noncoding RNA GSTM3TV2 upregulates LAT2 and OLR1 by competitively sponging let-7 to promote gemcitabine resistance in pancreatic cancer. J. Hematol. Oncol. 12, 97. 10.1186/s13045-019-0777-7 31514732 PMC6739963

[B158] Xiong J. Wang D. Wei A. Ke N. Wang Y. Tang J. (2017). MicroRNA-410-3p attenuates gemcitabine resistance in pancreatic ductal adenocarcinoma by inhibiting HMGB1-mediated autophagy. Oncotarget 8, 107500–107512. 10.18632/oncotarget.22494 29296182 PMC5746084

[B159] Xu C. Yu Y. Ding F. (2018). Microarray analysis of circular RNA expression profiles associated with gemcitabine resistance in pancreatic cancer cells. Oncol. Rep. 40, 395–404. 10.3892/or.2018.6450 29781033

[B160] Xu F F. Huang M. Chen Q. Niu Y. Hu Y. Hu P. (2021a). LncRNA HIF1A-AS1 promotes gemcitabine resistance of pancreatic cancer by enhancing glycolysis through modulating the AKT/YB1/HIF1α pathway. Cancer Res. 81, 5678–5691. 10.1158/0008-5472.CAN-21-0281 34593522

[B161] Xu F F. Wu H. Xiong J. Peng T. (2021b). Long non-coding RNA DLEU2L targets miR-210-3p to suppress gemcitabine resistance in pancreatic cancer cells via BRCA2 regulation. Front. Mol. Biosci. 8, 645365. 10.3389/fmolb.2021.645365 33968986 PMC8100451

[B162] Xu H. Chen R. Shen Q. Yang D. Peng H. Tong J. (2021). Overexpression of circular RNA circ_0013587 reverses erlotinib resistance in pancreatic cancer cells through regulating the miR-1227/E-cadherin pathway. Front. Oncol. 11, 754146. 10.3389/fonc.2021.754146 34552882 PMC8450525

[B163] Xu J. Wang T. Cao Z. Huang H. Li J. Liu W. (2014). MiR-497 downregulation contributes to the malignancy of pancreatic cancer and associates with a poor prognosis. Oncotarget 5, 6983–6993. 10.18632/oncotarget.2184 25149530 PMC4196178

[B164] Xu W. Zhou G. Wang H. Liu Y. Chen B. Chen W. (2020). Circulating lncRNA SNHG11 as a novel biomarker for early diagnosis and prognosis of colorectal cancer. Int. J. Cancer 146, 2901–2912. 10.1002/ijc.32747 31633800

[B165] Xu Y. (2023). MiRNA-21-5p accelerates EMT and inhibits apoptosis of laryngeal carcinoma via inhibiting KLF6 expression. Biochem. Genet. 61, 101–115. 10.1007/s10528-022-10246-z 35761154

[B166] Xu Y. Qin Y. Cui J. X. Xu J. (2020). MicroRNA-136-5p regulates gemcitabine resistance in pancreatic cancer via down-regulating ZNF32. Eur. Rev. Med. Pharmacol. Sci. 24, 10472–10482. 10.26355/eurrev_202010_23400 33155203

[B167] Xu Y. Yao Y. Gao P. Cui Y. (2019). Upregulated circular RNA circ_0030235 predicts unfavorable prognosis in pancreatic ductal adenocarcinoma and facilitates cell progression by sponging miR-1253 and miR-1294. Biochem. Biophys. Res. Commun. 509, 138–142. 10.1016/j.bbrc.2018.12.088 30591218

[B168] Yan H. J. Liu W. S. Sun W. H. Wu J. Ji M. Wang Q. (2012). miR-17-5p inhibitor enhances chemosensitivity to gemcitabine via upregulating Bim expression in pancreatic cancer cells. Dig. Dis. Sci. 57, 3160–3167. 10.1007/s10620-012-2400-4 23001407

[B169] Yang D. Hu Z. Xu J. Tang Y. Wang Y. Cai Q. (2019). MiR-760 enhances sensitivity of pancreatic cancer cells to gemcitabine through modulating Integrin β1. Biosci. Rep. 39. 10.1042/BSR20192358 PMC6863763 31693728

[B170] Yang F. Li X. Zhang L. Cheng L. Li X. (2018). LncRNA TUG1 promoted viability and associated with gemcitabine resistant in pancreatic ductal adenocarcinoma. J. Pharmacol. Sci. 137, 116–121. 10.1016/j.jphs.2018.06.002 29960845

[B171] Yang K. D. Wang Y. Zhang F. Li Q. L. Luo B. H. Feng D. Y. (2022). CAF-derived midkine promotes EMT and cisplatin resistance by upregulating lncRNA ST7-AS1 in gastric cancer. Mol. Cell Biochem. 477, 2493–2505. 10.1007/s11010-022-04436-x 35588343

[B172] Yang Q. Li K. Huang X. Zhao C. Mei Y. Li X. (2020). lncRNA SLC7A11-AS1 promotes chemoresistance by blocking SCFβ-TRCP-mediated degradation of NRF2 in pancreatic cancer. Mol. Ther. Nucleic Acids 19, 974–985. 10.1016/j.omtn.2019.11.035 32036249 PMC7013141

[B173] Yang R. M. Zhan M. Xu S. W. Long M. M. Yang L. H. Chen W. (2017). miR-3656 expression enhances the chemosensitivity of pancreatic cancer to gemcitabine through modulation of the RHOF/EMT axis. Cell Death Dis. 8, e3129. 10.1038/cddis.2017.530 29048402 PMC5682692

[B174] Yang S. Z. Xu F. Zhou T. Zhao X. Mcdonald J. M. Chen Y. (2017). The long non-coding RNA HOTAIR enhances pancreatic cancer resistance to TNF-related apoptosis-inducing ligand. J. Biol. Chem. 292, 10390–10397. 10.1074/jbc.M117.786830 28476883 PMC5481552

[B175] Yang Z. Zhao N. Cui J. Wu H. Xiong J. Peng T. (2020). Exosomes derived from cancer stem cells of gemcitabine-resistant pancreatic cancer cells enhance drug resistance by delivering miR-210. Cell Oncol. (Dordr) 43, 123–136. 10.1007/s13402-019-00476-6 31713003 PMC12990725

[B176] Yao J. Li Z. Wang X. Xu P. Zhao L. Qian J. (2016). MiR-125a regulates chemo-sensitivity to gemcitabine in human pancreatic cancer cells through targeting A20. Acta Biochim. Biophys. Sin. (Shanghai) 48, 202–208. 10.1093/abbs/gmv129 26758190

[B177] Ye X. Wang L. P. Han C. Hu H. Ni C. M. Qiao G. L. (2022). Increased m(6)A modification of lncRNA DBH-AS1 suppresses pancreatic cancer growth and gemcitabine resistance via the miR-3163/USP44 axis. Ann. Transl. Med. 10, 304. 10.21037/atm-22-556 35433957 PMC9011309

[B178] Ye Z. Q. Chen H. B. Zhang T. Y. Chen Z. Tian L. Gu D. N. (2021). MicroRNA-7 modulates cellular senescence to relieve gemcitabine resistance by targeting PARP1/NF-κB signaling in pancreatic cancer cells. Oncol. Lett. 21, 139. 10.3892/ol.2020.12400 33552258 PMC7798037

[B179] Ye Z. Q. Zou C. L. Chen H. B. Jiang M. J. Mei Z. Gu D. N. (2020). MicroRNA-7 as a potential biomarker for prognosis in pancreatic cancer. Dis. Markers 2020, 2782101. 10.1155/2020/2782101 32566037 PMC7288197

[B180] Yin F. Zhang Q. Dong Z. Hu J. Ma Z. (2020). LncRNA HOTTIP participates in cisplatin resistance of tumor cells by regulating miR-137 expression in pancreatic cancer. Onco Targets Ther. 13, 2689–2699. 10.2147/OTT.S234924 32280243 PMC7132030

[B181] You L. Chang D. Du H. Z. Zhao Y. P. (2011). Genome-wide screen identifies PVT1 as a regulator of Gemcitabine sensitivity in human pancreatic cancer cells. Biochem. Biophys. Res. Commun. 407, 1–6. 10.1016/j.bbrc.2011.02.027 21316338

[B182] Yu C. Wang M. Chen M. Huang Y. Jiang J. (2015). Upregulation of microRNA1385p inhibits pancreatic cancer cell migration and increases chemotherapy sensitivity. Mol. Med. Rep. 12, 5135–5140. 10.3892/mmr.2015.4031 26135834

[B183] Yu G. Jia B. Cheng Y. Zhou L. Qian B. Liu Z. (2017). MicroRNA-429 sensitizes pancreatic cancer cells to gemcitabine through regulation of PDCD4. Am. J. Transl. Res. 9, 5048–5055. 29218103 PMC5714789

[B184] Yu Q. Xiu Z. Jian Y. Zhou J. Chen X. Chen X. (2022). microRNA-497 prevents pancreatic cancer stem cell gemcitabine resistance, migration, and invasion by directly targeting nuclear factor kappa B 1. Aging (Albany NY) 14, 5908–5924. 10.18632/aging.204193 35896012 PMC9365558

[B185] Yu S. Li Y. Liao Z. Wang Z. Wang Z. Li Y. (2020). Plasma extracellular vesicle long RNA profiling identifies a diagnostic signature for the detection of pancreatic ductal adenocarcinoma. Gut 69, 540–550. 10.1136/gutjnl-2019-318860 31562239

[B186] Yu S. Wang M. Zhang H. Guo X. Qin R. (2021). Circ_0092367 inhibits EMT and gemcitabine resistance in pancreatic cancer via regulating the miR-1206/ESRP1 Axis. Genes (Basel) 12, 1701. 10.3390/genes12111701 34828307 PMC8622583

[B187] Yu Y. Zou Y. F. Hong R. Q. Chen W. J. Chen L. Chen W. Q. (2022). Long non-coding RNA SNHG16 decreased SMAD4 to induce gemcitabine resistance in pancreatic cancer via EZH2-mediated epigenetic modification. Kaohsiung J. Med. Sci. 38, 981–991. 10.1002/kjm2.12574 36053032 PMC11896543

[B188] Zeng Z. Zhao Y. Chen Q. Zhu S. Niu Y. Ye Z. (2021). Hypoxic exosomal HIF-1α-stabilizing circZNF91 promotes chemoresistance of normoxic pancreatic cancer cells via enhancing glycolysis. Oncogene 40, 5505–5517. 10.1038/s41388-021-01960-w 34294845

[B189] Zhan T. Chen X. Tian X. Han Z. Liu M. Zou Y. (2020). MiR-331-3p links to drug resistance of pancreatic cancer cells by activating WNT/β-Catenin signal via ST7L. Technol. Cancer Res. Treat. 19, 1533033820945801. 10.1177/1533033820945801 32924881 PMC7493267

[B190] Zhang H. Deng T. Liu R. Ning T. Yang H. Liu D. (2020). CAF secreted miR-522 suppresses ferroptosis and promotes acquired chemo-resistance in gastric cancer. Mol. Cancer 19, 43. 10.1186/s12943-020-01168-8 32106859 PMC7045485

[B191] Zhang L. Yao J. Li W. Zhang C. (2018). Micro-RNA-21 regulates cancer-associated fibroblast-mediated drug resistance in pancreatic cancer. Oncol. Res. 26, 827–835. 10.3727/096504017X14934840662335 28477403 PMC7844724

[B192] Zhang Q. A. Yang X. H. Chen D. Yan X. Jing F. C. Liu H. Q. (2018). miR-34 increases *in vitro* PANC-1 cell sensitivity to gemcitabine via targeting Slug/PUMA. Cancer Biomark. 21, 755–762. 10.3233/CBM-170289 29355113 PMC13078340

[B193] Zhang X. Zhao P. Wang C. Xin B. (2019). SNHG14 enhances gemcitabine resistance by sponging miR-101 to stimulate cell autophagy in pancreatic cancer. Biochem. Biophys. Res. Commun. 510, 508–514. 10.1016/j.bbrc.2019.01.109 30737032

[B194] Zhang X. Zheng S. Hu C. Li G. Lin H. Xia R. (2022). Cancer-associated fibroblast-induced lncRNA UPK1A-AS1 confers platinum resistance in pancreatic cancer via efficient double-strand break repair. Oncogene 41, 2372–2389. 10.1038/s41388-022-02253-6 35264742 PMC9010302

[B195] Zhang Y. Han T. Li J. Cai H. Xu J. Chen L. (2020). Comprehensive analysis of the regulatory network of differentially expressed mRNAs, lncRNAs and circRNAs in gastric cancer. Biomed. Pharmacother. 122, 109686. 10.1016/j.biopha.2019.109686 31786464

[B196] Zhao B. Song X. Guan H. (2020). CircACAP2 promotes breast cancer proliferation and metastasis by targeting miR-29a/b-3p-COL5A1 axis. Life Sci. 244, 117179. 10.1016/j.lfs.2019.117179 31863774

[B197] Zhao L. Zou D. Wei X. Wang L. Zhang Y. Liu S. (2016). MiRNA-221-3p desensitizes pancreatic cancer cells to 5-fluorouracil by targeting RB1. Tumour Biol. 37, 16053–16063. 10.1007/s13277-016-5445-8 27726102

[B198] Zhao Y. Zhao L. Ischenko I. Bao Q. Schwarz B. Niess H. (2015). Antisense inhibition of microRNA-21 and microRNA-221 in tumor-initiating stem-like cells modulates tumorigenesis, metastasis, and chemotherapy resistance in pancreatic cancer. Target Oncol. 10, 535–548. 10.1007/s11523-015-0360-2 25639539

[B199] Zhou C. Yi C. Yi Y. Qin W. Yan Y. Dong X. (2020). LncRNA PVT1 promotes gemcitabine resistance of pancreatic cancer via activating Wnt/β-catenin and autophagy pathway through modulating the miR-619-5p/Pygo2 and miR-619-5p/ATG14 axes. Mol. Cancer 19, 118. 10.1186/s12943-020-01237-y 32727463 PMC7389684

[B200] Zhou L. Jia S. Ding G. Zhang M. Yu W. Wu Z. (2019). Down-regulation of miR-30a-5p is associated with poor prognosis and promotes chemoresistance of gemcitabine in pancreatic ductal adenocarcinoma. J. Cancer 10, 5031–5040. 10.7150/jca.31191 31602254 PMC6775620

[B201] Zhu H. Shan Y. Ge K. Lu J. Kong W. Jia C. (2020). LncRNA CYTOR promotes pancreatic cancer cell proliferation and migration by sponging miR-205-5p. Pancreatology 20, 1139–1148. 10.1016/j.pan.2020.05.004 32732173

[B202] Zhu X. X. Li J. H. Ni X. Wu X. Hou X. Li Y. X. (2022). Pancreatic ductal adenocarcinoma cells regulated the gemcitabine-resistance function of CAFs by LINC00460. Cancer Sci. 113, 3735–3750. 10.1111/cas.15547 36047966 PMC9633316

